# Reasoning about possibilities: Modal logics, possible worlds, and mental models

**DOI:** 10.3758/s13423-024-02518-z

**Published:** 2024-07-16

**Authors:** P. N. Johnson-Laird, Marco Ragni

**Affiliations:** 1 Department of Psychology, Princeton University, Princeton, NJ 08540 USA; 2 Department of Psychology, New York University, 6 Washington Place, New York, NY 10003 USA; 3 Technische Universität Chemnitz, Thüringer Weg 11, 09126 Chemnitz, Germany

**Keywords:** Logical validity, Mental models, Modal logics, Necessity, Possible worlds, Reasoning

## Abstract

Everyone reasons about possibilities. This article explains how they could do so using mental models. The theory makes four major claims: 1. Correct inferences are necessary, referring only to facts or possibilities to which the premises refer and not ruling any of them out, for example: *She left or hid*; Therefore, *it’s possible that she left* and *possible that she hid*. 2. A possibility such as that she hid, which is represented in an intuitive model, presupposes the possibility that it did not occur, *she did not hide*, which, if reasoners deliberate, is represented in the resulting model. 3. Reasoners condense consistent possibilities, such as the earlier pair, into one possibility: *it is possible that she left and she hid*. 4. Inconsistencies, such as *she left or hid, and she neither left nor hid*, refer to no possibilities whatsoever – they have an empty model – and so their only effects are local. Hence, any inference can be withdrawn with impunity if there is knowledge to the contrary. Experiments have corroborated each of these principles. They are incompatible with four essentials of standard modal logics, which concern deductions based on “possible” or “necessary”. Their formal deductions correspond to valid inferences, which have no counterexamples in which the premises are true but the conclusion is false. And so the article examines the differences between the two approaches, and explores the adaptation of a modal logic to account for correct human reasoning. Its feasibility is an open question.

**Supplementary Information:**

The online version contains supplementary material available at 10.3758/s13423-024-02518-z.

## Introduction

How did it come to be that logic which, at least in the views of some people 2300 years ago, was supposed to deal with evaluations of argumentation in natural languages, has done a lot of extremely interesting and important things, but not this?—Bar-Hillel, 1969

The point of logic is to give an account of the notion of validity: what follows from what.—Priest, 2008

In his *Theodicy*, Leibniz (1985/1710) wrote that God made for us the best of all possible worlds. To which Voltaire asked, in the character of his Holy innocent, Candide: “If this is the best of all possible worlds what are the others like?” Leibniz’s concept of possible worlds, however, was influential. It is applicable to *modal* assertions about possibilities, such as:

- *Possibly the stock-market will fall tomorrow.*

Modal logics deal with such sentences, and treat them as true if there is a possible world relevant to the truth values of sentences about the real world in which the stock-market does fall tomorrow, but false if there is no such possible world. For readers unfamiliar with modal logics, Appendix 1 outlines their general principles and the semantics of possible worlds. Aristotle, the founder of Western logic, analyzed modal inferences. And he made a pertinent observation:

The negation of “possible not to be” is “not posssible not to be”. That is why “possible to be” and “possible not to be” may be thought actually to follow from one another. (*De Interpretatione,* Ch. 12, lines 34-36, in Barnes, 1984.)

If readers find his second claim obvious, that is the point. They will see presently why it matters.

The purpose of logic for Aristotle was to guide correct reasoning and thereby to improve scientific knowledge. But the great burgeoning of logic from the second half of the nineteenth century onwards in the works of Frege, Russell, Hilbert, and others, aimed instead to prove that arithmetic is nothing more than the logic of natural numbers (0, 1, 2, 3, …). This goal had no need for modal logics, but it failed when Gödel (1965/1931) proved his first “incompleteness” theorem. He formulated a sentence asserting its own unprovability, encoded it as a natural number, and showed that neither it nor its negation could be proved in a (strongly) consistent formal logic for the arithmetic of natural numbers. Yet the sentence is obviously true for us humans, which led Gödel and others to conclude that human thinking cannot be algorithmic (see Johnson-Laird, Byrne, & Khemlani, 2023). Modal logics burgeoned later thanks to Kripke’s (1963) innovative semantics for them using relations between possible worlds (see Appendix 1). These logics are studied for several reasons – for their intrinsic interest, for a metaphysics of possible worlds (e.g., Lewis, 1986), for the “many worlds” interpretation of quantum mechanics (Everett, 1957), for the semantics of English as a formal language (Montague, 1974), for the analysis of provability (Boolos & Sambin, 1991), and for accounts of the meanings of assertions in natural language (e.g., Chung & Mascarenhas, 2023; Lewis, 1973; Stalnaker, 1968). The latter use does not imply that modal logic explains human reasoning, though some cognitive psychologists, as we show, have treated it in this way. Our theory of modal reasoning is based on mental models but it owes much to modal logics, and the present article reciprocates. It presents some ways to modify modal logic so it might serve as a basis for a cognitive theory of reasoning.

Psychologists have made many empirical investigations of possibility, but with a focus on children’s grasp of the concept (e.g., Byrnes & Beilin, 1991; Inhelder & Piaget, 1958; Piéraut-Le Bonniec, 1980; Shtulman & Phillips, 2018). One study examined modal conclusions inferred from categorical premises (Evans et al., 1999). And the pioneering study is due to the late Daniel Osherson (1976). He tested whether a simple modal logic (which he devised as a subset of one known as system T, see Appendix 1) could be the basis of human reasoning. His participants had to assess whether given inference followed, and to put into rank order of difficulty all those that they judged to follow. He tested only inferences valid in his logic, such as the following examples, where *A* and *B* were assertions about lights that were bright or dim, on or off, etc.:

- *Necessarily (A and B). * $\therefore$  *Necessarily A and necessarily B.*
- *Necessarily not A. * $\therefore$  *Not possibly A.*

The symbol ‘∴’ stands for “therefore”. Osherson sought to fit his model of the difficulty of inferences to the data. But the result was “not entirely satisfactory” (ibid., p. 232). For instance, many participants failed to accept the second inference above. Perhaps another modal logic would have yielded a better fit. But it is unlikely, because the second inference is valid in all the countable infinity of modal logics in which the two modal operators, *possible* and *necessary*, are interdefinable:

- *Possible* is equivalent to *Not necessarily not the case*.
- *Necessary* is equivalent to *Not possibly not the case*.

The present article concerns human reasoning about possibilities. Its standpoint is the theory of mental models – the “model theory,” for short. Elsewhere its authors have argued that three sorts of possibility occur in daily life: *epistemic* possibilities based on empirical knowledge, *deontic* possibilities based on knowledge of what is permissible or obligatory, and *alethic* possibilities based on the relations between propositions or between concepts (Johnson-Laird & Ragni, 2019; Ragni & Johnson-Laird, 2020). The latter study also presented syntactic and contextual cues that elicit one interpretation of a possibility rather than another. Elsewhere, too, are studies of deontic reasoning (e.g., Bucciarelli & Johnson-Laird, 2005, 2019; Bucciarelli, Khemlani, & Johnson-Laird, 2008). But we have focused this article on a self-contained examination of everyday inferences about epistemic possibilities. In daily life they range from impossibility to barely possible, and thence to possible, and so on up to certainty in contrast to the necessity of modal logics (see, e.g., Lassiter, 2017; White, 1975). The proportions of models supporting a possible conclusion provide a gateway into probabilistic reasoning (Johnson-Laird & Ragni, 2019), which treats subjective probabilities either as non-numerical degrees of intuitive possibility, or, with deliberation, as a numerical scale ranging from, say, 0% to 100% (Khemlani, Lotstein, & Johnson-Laird, 2015).

One other major sort of psychological theorizing about reasoning exists. It relates to conceptions of probability due to Ramsey (1990/1926), de Finetti (1995/1936), and Adams (1998). And it defends various sorts of probabilistic accounts of inferences (e.g., Cruz et al., 2015; Douven et al., 2018; Fugard et al., 2011; Over et al., 2007; Oaksford & Chater, 2007, 2020). These probabilists should be credited for the first attempt to explain human reasoning without relying on logic. We consider their accounts only in passing, because they do not deal with modal assertions, and because there is an alternative, but corroborated, psychological theory of probabilities founded on mental models (Goodwin, 2014; Johnson-Laird & Khemlani, 2022; Khemlani et al., 2015a, 2015b).

Our article begins with four essential principles of standard modal logics. And for readers unfamiliar with modal logics, Appendix 1 describes how they work. The four principles organize the rest of the article. It outlines the original theory of mental models, and Appendix 2 surveys the processes that construct them and draw conclusions from them. The article then describes the current model theory of modal reasoning, and accompanies it with corroboratory evidence. The results conflict with the essential principles of standard modal logics. Next the article takes these principles into account to assess the feasibility of a cognitive theory of reasoning that embodies a modal logic. The article ends with a discussion of the principal advantages that mental models confer on human reasoning.

## Four essentials of standard modal logics

The classical sentential calculus deals with the logic of negation, *not*, and of sentential connectives, such as the logical doppelgangers of *if*, *or*, and *and*, which have fixed meanings that are idealized modifications of their everyday interpretations. It treats every sentence as either true or false, and the truth values of sentences formed with connectives, i.e., “compound” sentences, as depending solely on those of their constituent clauses. For example, a disjunction of two clauses such as: *It’s raining or it’s freezing or both*, is true if at least one of its two clauses is true, and false only if both of its clauses are false. We define a *standard* logic as one that includes the sentential calculus, that is:

- the sentential calculus itself,
- the predicate calculi dealing in addition with the quantifiers *some*, *all*, and more complex quantifiers such as *most*,
- modal logics dealing with *possible* and *necessary*, incorporated within the sentential calculus or a predicate calculus,
- the logic for arithmetic, as in Gödel’s proof.

We now consider four essential principles of standard modal logics.

## Validity

The first essential is that validity is the criterion for correct reasoning, as in the epigraph to the present paper from Priest (2008). An inference is defined as *valid* in case its conclusion is true in every case in which its premises are true (Jeffrey, 1981, p. 1). It therefore has no counterexample in which its premises are true but its conclusion is false: validity preserves truth.

## Facts imply possibilities

The second essential of standard modal logics – including system T, which Osherson (1976) studied, and which is embodied in many others – is that they have a formal rule of inference (or equivalent axiom) in which a categorical assertion, such as:

- *It was hot. * [A]

implies the corresponding possibility:

- $\therefore$ *Possibly it was hot.  * [$\therefore$ Possibly A]

Formal proofs operate as specified manipulations of symbols without regard to their meanings, but any standard logic, such as system T, has a separate semantics, such as one based on “possible worlds”. It assigns one of two truth values, *true* or *false*, to sentences, and it ensures that the inference above is valid.

## Invalidity of condensations of possibilities

The third essential of all standard modal logics is that any inference that condenses a conjunction of two possibilities into one possibility is invalid. Consider, for example, the following inference:

- *     It’s possible that it’s sunny and it’s possible that it’s hot.* [Possibly A and possibly B]
- $\therefore$ *It’s possible that it’s sunny and that it’s hot.*  [$\therefore$  Possibly (A and B)]

Despite its plausibility, it is invalid because one clause in the premise could hold in one possible world and the other clause in another possible world. The need for two possible worlds is obvious in the next example, because the two possibilities are contrary to one another, i.e., they cannot both be true:

- *Possibly the coin landed heads and possibly the coin landed tails.*

Their conjunction into a single possibility would produce an inconsistency. In contrast, the condensation of disjunctive possibilities is valid in all standard modal logics, whether the disjunction is “inclusive” in that it allows both clauses to be true, or exclusive in that it does not, as in this example:

- * Possibly it will be cloudy or possibly it will be cold, but not both.*
- $\therefore$ *Possibly it will be cloudy or cold, but not both*.

Readers who maintain an agnostic position about whether human reasoning depends on some sort of formal rules of inference or on mental models should note that a derivation of the preceding inference calls for some 40 formal steps in the simplest modal logic, system K, in part because an exclusive disjunction has to be represented as a conjunction of an inclusive disjunction with a denial of the conjunction in which both clauses hold. The need for such lengthy derivations makes it unlikely that human reasoners rely on them.

## Explosive inconsistencies

The fourth essential of standard modal logics is a direct consequence of the sentential calculus. Inconsistency in premises is a disaster in all standard logics, because it yields any conclusion whatsoever as a valid inference. An inconsistency means that the sentences cannot all be true (at the same time), and so one of them is false. It follows that there cannot be a counterexample in which all the premises are true (and the conclusion is false). For example, the following inference is valid in all standard modal logics:

- *   It is possible that the stock-market will fall tomorrow.*
- *  It is not possible that the stock-market will fall tomorrow.*
- $\therefore$ *A rhinoceros is in your bath.*

There can be no case in which the premises are true, and so the inference is bound to be valid. The formal rules of a standard modal logic also allow the following proof to be made, starting with the first premise:

- *It is possible that the stock-market will fall tomorrow.*

Its truth guarantees the truth of the following interim conclusion, and the proof uses the corresponding formal rule: *A*; $\therefore$ *It is possible that A or that B or both*, where *B* can be any sentence whatsoever:

$\therefore$ *It is possible that the stock-market will fall tomorrow or that a rhinoceros is in your bath, or both*.

Given the preceding disjunction, and the second premise:

- *It is not possible that the stock-market will fall tomorrow*

the other clause of the disjunction can be inferred using the formal rule:

- *     A or B or both*, *Not A*;  $\therefore$  *B*:
- $\therefore$ *A rhinoceros is in your bath.*  (QED)

The four essentials of modal logics conflict with mental models and their experimental corroborations. So a crucial task for any cognitivist seeking to build a theory based on a modal logic is to find a way to modify the logic. We will make some suggestions about how to do so later, but first we need to describe the model theory.

## The original theory of mental models

The initial conception of mental models is due to the Scottish psychologist Kenneth Craik (1943). He postulated that the human mind constructs “small-scale models” of reality that it runs faster than real time so that the resulting anticipations can guide decisions about future actions. Mental models, he claimed, have the same-input output relations as the entities they simulate but “…the model need not resemble the real object pictorially” (ibid. p. 51). His exemplar was Kelvin’s tide predictor, a machine using a crank and shafts geared together, but with no structural resemblance to the earth, moon, and tides. Humans do not use models for reasoning, because it depends on verbal “rules of implication” (ibid., p. 78-9).

Over 25 years later, an independent theory of mental models began as an explanation of how people understand discourse: they build small-scale models of the situations it describes (Garnham, 1987; Johnson-Laird, 1970). The theory postulated that a mental model is *iconic* in that its structure corresponds to that of the situation it represents insofar as possible. This assumption explains spatial reasoning: conclusions emerge from relations in an iconic model integrating separate assertions (e.g., Johnson-Laird, 1972; Ragni & Knauff, 2013). It also explains syllogistic reasoning in which models containing sets of tokens represent actual sets of entities from which new conclusions can emerge (Johnson-Laird, 1975; Johnson-Laird & Steedman, 1978; Khemlani & Johnson-Laird, 2022). Readers unfamiliar with the model theory should consult Appendix 2 for how sets of models from different premises are conjoined, how they represent epistemic possibilities, and how they represent sets of entities.

The model theory makes background assumptions about what has to be computed in a deduction. Namely, conclusions should not contain less semantic information than their premises, they should be more parsimonious than their premises but can take for granted categorical premises, and they should state something new, which is not explicit in the premises themselves (Johnson-Laird, 1983, p. 37 et seq.). Reasoners are unlikely to be aware of these principles or even to rely on them. They are instead emergent properties from the use of models in making inferences. Indeed, the original theory’s principal corroborations were in explaining how individuals can draw their own conclusions from premises, why they are faster and more accurate when the premises yield only a single model as opposed to multiple models, and why the latter sometimes elicit the response that nothing follows from the premises. For example, the models of the premises: *None of the beekeepers is an artist and none of the beekeepers is a chemist* include those in which *no artists are chemists*, and those in which *some artists are chemists*. Hence both are possibilities, but no categorical conclusion holds in all the models, and so reasoners judge that nothing follows (see Johnson-Laird, 1983, Ch. 5, for a review). These results go beyond what standard logics themselves can explain, because these logics allow infinitely many valid conclusions to follow from any premises, for example, a conjunction of a premise with itself, or an inclusive disjunction of a premise with any other assertion. Hence, logic has no way to predict which conclusions human reasoners tend to draw from among the infinity. Yet the original model theory was consistent with standard logics, and so it offered a psychologically plausible semantics for logic (Johnson-Laird, 1983). It allowed, however, that pragmatic principles of the sort due to Grice (1975) were crucial in reasoners’ formulation of conclusions (ibid, p. 38-9). The theory led to an accumulation of experimental results establishing that human reasoners do not use formal rules of inference to reason, but instead envisage models of the premises and infer conclusions that hold in all of them (Johnson-Laird & Byrne, 1991).

The implementation of the theory in a computer program led to the discovery of compelling and predictable inferences that are fallacious (Johnson-Laird & Savary, 1999) – they seemed at first to reflect a bug in the program, but the bug was in the programmer’s mind. We refer to them as “illusory” inferences, because nearly everyone makes them with great confidence. Yet they are fallacious. Here is an example:

- *Either Jane is kneeling by the fire and she is looking at the TV or otherwise*
- *Mark is standing at the window and he is peering into the garden.*
- *Jane is kneeling by the fire.*
- *Does it follow that she is looking at the TV?*

The intuitive models of the first premise represent two possibilities, which we abbreviate in a diagram following the convention that each alternative model is represented on a separate line:

- Jane: kneeling looking
-                                     Mark: standing peering

Of course, real mental models are hardly made out of words, which we use for convenience. In reality, they are iconic representations akin to perceptions of scenes. The second premise picks out the first possibility, and so it predicts an affirmative response. Most participants did indeed respond: “Yes” (Walsh & Johnson-Laird, 2004). In fact, the disjunction could be true because its second clause is true, but its first clause is false – in which case, it does not follow that Jane is looking at the TV even though she is kneeling by the fire. The correct answer is therefore: “No”. An alternative account of this illusory inference is due to Koralus and Mascarenhas (2013). Many other sorts of predicted illusory inferences occur, and participants tend to make correct control inferences with similar contents (e.g., Ragni, Sonntag, & Johnson-Laird, 2016; for a review, see Khemlani & Johnson-Laird, 2017). The discovery of these fallacies was a further corroboration that human reasoners do not rely on formal rules of inference. Formal rules for them would be inconsistent with formal rules for valid inferences, and lead to chaos.

Extensions to the theory explained certain inductive and abductive inferences (e.g., Johnson-Laird, 2006; Johnson-Laird, Girotto, & Legrenzi, 2004; Khemlani et al., 2013). And Koralus and Mascarenhas (2013) developed their own version of the model theory, which is consistent with the formal semantics of standard logics (see Koralus, 2023). It treats reasoning as a sequence of answers to the questions that premises raise, for example, a disjunctive premise such as “Ann is in Paris or she is in Rome” raises the question of which city she is in. So, as reasoners cope with each new premise, their answers yield valid conclusions. This “erotetic” theory often runs in parallel with the model theory, but there are differences between them, which may be larger in the current version of the model theory, which is the topic of the next section, and which explains reasoning about possibilities.

## The model theory of modals and its corroborations

The current model theory maintains many of the principles of its precursors. It still distinguishes between immediate intuitive models and the subsequent option of deliberative models. Intuitive models are constructed with no use of working memory for intermediate results, and each model represents a distinct possibility and within it only those clauses in the premises that hold in the possibility. Deliberative models, and the thoughts yielding them, can use working memory for intermediate results. They also represent clauses from the premises that do not hold in a possibility, and they use negation to do so. Of course, any model is itself held in working memory, but only deliberation can manipulate models there. Wason (1966) pioneered such dual systems for reasoning, and its first algorithm was for his well-known task of selecting evidence to find out whether a conditional hypothesis is true (Johnson-Laird & Wason, 1970). Its actual computer implementation and a meta-analysis of over 200 experiments is much more recent (Ragni, Kola, & Johnson-Laird, 2018). Several theorists have adopted such “dual systems”, for example, Evans (2008) and De Neys (2023), and they were made famous in the late Danny Kahneman’s (2011) brilliant best-selling book. The cause of the illusory inferences described earlier is that intuitive models represent only what is the case, but deliberative models can correct resulting errors.

The fundamental assumption of the current model theory is that human reasoning is founded on possibilities, and that each mental model represents what is common to all the different ways in which a possibility can occur. For instance, the possible outcomes of a coin toss have three models (head, tails, and neither heads nor tails). Each model captures what is common to all the different spins, tosses, coins, etc. in tosses in which the outcome could occur. There are analogous conceptions of possible worlds as small sets of assertions or as miniworlds rather than Leibnizian maxiworlds akin to planets (see Appendix 1). The present section finesses the details of the processes that construct, modulate, and interpret models, but they are described in Appendix 2 below.

Disjunctions are rooted in the ability to grasp that in any situation, mutually exclusive and exhaustive alternatives can occur (Johnson-Laird & Ragni, 2019). Great apes never develop this ability, but 3-year-old children acquire it (Redshaw et al., 2019). Both inclusive and exclusive disjunction can express such primordial alternatives, but they have other interpretations in which they do not, which we consider later. The primordial interpretation of a disjunction refers to a conjunction of possibilities that each hold in default of knowledge to the contrary, though at least one must hold for the disjunction to be true. The following disjunction about, say, shapes on a whiteboard:

There is a square or a circle, or both

has a primordial meaning, referring to a conjunction of possibilities. Intuition constructs models of these possibilities one at a time, and the diagram of them below uses again the convention that each model is represented on a separate line:
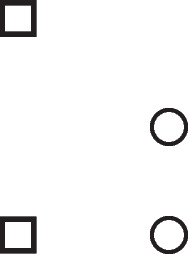


Deliberative models represent in addition those clauses in the premises that do not hold in a possibility, using negation to do so in the models:
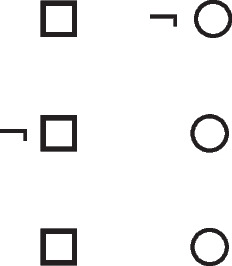


where ‘¬’ is symbol denoting negation. Both sets of models are in a conjunction, and each model hold in default of knowledge contrary to the possibility that it represents. So, both the third intuitive model and the third deliberative model imply:

- $\therefore$ *It is possible that there is a square and a circle.*

Most participants in an experiment judged that such conclusions from everyday disjunctions do follow (Hinterecker, Knauff, & Johnson-Laird, 2016). They also judged that the possibility of each individual clause follows too, rejecting only the possibility that denies both clauses in a disjunction. These inferences follow in the model theory, because their conclusions refer only to possibilities to which their premise refers. The model theory postulates that all inferences hold only in default of knowledge to the contrary, and the present inferences are no exception. It could be impossible that there is a square but that there is a circle. This situation is a counterexample to the inference above, which explains why it is invalid in standard logics. The discovery that the conclusion is, in fact, false would justify its withdrawal and elimination of the two models referring to a square from the sets above. Conditionals have analogous models, but conditionals are special, and so we describe their models in a later section on presupposed possibilities and conditionals. Knowledge and meaning can *modulate* the interpretation of assertions, blocking the construction of a model of a possibility, or adding information to a model (Johnson-Laird & Byrne, 2002). The model theory assumes that knowledge is represented in deliberative models, but how the process of understanding a premise triggers such models calls for a major research project. In our program simulating modulation, we have used a simplistic process that merely searches for a match between lexical items in a sentence and those in the knowledge base (see mSentential at https://www.modeltheory.org/models/). The use of Large Language Models in AI suggests that simple procedures writ large can be surprisingly effective. Once appropriate knowledge is retrieved from long-term memory, a conjunction is made of its set of models with those of the assertion.

Modulation can affect inferences. For example, individuals are likely to recognize that the following inference follows (Johnson-Laird, 1969):

- * She died and she took the medicine.*
- $\therefore$ *She took the medicine and later died.*

and that the following conjunction is not a contradiction:

- *He likes gin and he likes tonic but he doesn’t like gin and tonic.*

Modulation yields various interpretations of disjunctions (Quelhas & Johnson-Laird, 2017) and conditionals (Johnson-Laird & Byrne, 2002; Quelhas, Johnson-Laird, & Juhos, 2010). For example, given a disjunction such as:

*Miguel is at the beach or he is at home.*

participants know that one person cannot be in both places at the same time. So, if the disjunction is true, they judge that there are only two possibilities for Miguel’s whereabouts:
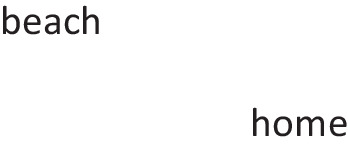


They tend to make the appropriate inferences from such disjunctions (Quelhas & Johnson-Laird, 2017). Modulation can be unconscious, for example, individuals adjust the tense of a verb from present to past depending on the temporal order of events, without awareness that they are doing so (Juhos, Quelhas, & Johnson-Laird, 2012).

Natural languages include “true”, “false”, and other ways to refer to truth values. Unlike logic, these expressions are not segregated within a meta-language for formulating the semantics of the language in which proofs are carried out. The consequences are twofold (Johnson-Laird et al., 2023). On the one hand, it is easy to express semantic paradoxes in everyday language, for example:

This sentence is false.

The sentence is a version of the well-known “liar” paradox, which refers to itself. So, if is true, then it follows that it is false, and vice versa. It is a genuine paradox. On the other hand, truth values in daily life can be richer than those in logic, such as:

- *It could be true and it could be false.*
- *It’s true and it couldn’t be false.*
- *It’s neither true nor false.*

Only non-standard logics recognize that sentences can be neither true nor false (Priest, 2008).

We now describe four essential principles of the model theory, and their experimental corroborations. Each of them is contrary to a corresponding essential principle of standard modal logics. Table 1 below should help readers to keep track of the argument.

## Correct inferences are alethic necessities

The model theory postulates that reasoners tacitly use the alethic category of necessity to assess whether an inference is correct. Aristotle also argued that inferences should be necessary (*Prior Analytics*, Ch. 1, line 24b20 in Barnes, 1984, Vol. 2), but he took its meaning for granted. Logicians sometimes refer to “necessary inferences” in the sense that the truth of the premises necessitates the truth of the conclusion, i.e., as a synonym for validity. Logicians tend to define validity as exemplified here:

“A valid inference is one whose conclusion is true in every case in which all its premises are true” (Jeffrey, 1981, p. 1)

We likewise define a necessary inference as follows:

A necessary inference is one whose conclusion only refers to one or more of the possibilities or facts to which its premises refer, and does not deny any of them.

In other words, it preserves possibilities (and facts). As Jeffrey remarked, the difficulty in applying the definition of validity comes from canvassing all the cases mentioned in it. Likewise, the difficulty in applying the definition of necessity comes from determining the possibilities that it concerns. One difficulty is that factual consequences must hold in all models of the premises, and so premises can have factual consequences. The solutions to other difficulties are illustrated in the following output of a program (mSentential) that assesses the alethic status of conclusions. A premise of the sort, *A or B but not both*, yields the following deliberative models of the possibilities to which it refers:
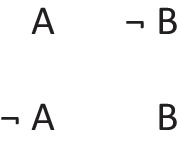


The principal alethic relations between premise models and conclusion models yield the following assessments:

1. The conclusion: *If and only if A then not B*, is necessary, because its models of possibilities are identical to those of the premise.

2. The conclusion: *Possibly B* is necessary because its model and the one for its presupposition (*Possibly not B*) both occur in the models of the premises.

3. The conclusion: *A or B, or both* does not follow of necessity, but only as an alethic possibility, because it has a model of a possibility that the premise does not have:
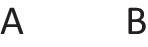


4. The conclusion, *Possibly B and not possibly A*, is not possible because it denies a possibility to which the premise refers.

5. The conclusion: *Either A or else not B* is not possible because its deliberative models:
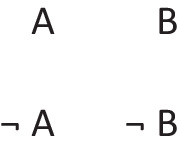


are inconsistent with those of the premise.

6. The conclusion: *C and D* is independent of the models, because they have no element in common.

Necessity and validity overlap: many inferences are both necessary and valid (as 1 above), some are necessary but not valid (as 2), some are valid but not necessary (as 3), and some are neither necessary nor valid (as 3, 4, and 5).

Reasoners who are assessing whether inferences follow from premises tend to reject those that are not necessary but only alethically possible. This claim is corroborated in an inference of this sort:

- * Ana read Don Quixote.*
- $\therefore$ *Ana read Don Quixote or a newspaper, or both.*

Nothing in the premise refers to the possibility that Ana read a newspaper, so the inference is not necessary but it is valid: the participants in an experiment rejected it (Orenes & Johnson-Laird, 2012). An illuminating contrast occurred with this inference:

-     *Ana read Don Quixote.*
- $\therefore$ *Ana read Don Quixote or a novel, or both.*

The participants in the experiment were Spanish, and they knew that *Don Quixote* is Cervantes’s great novel. So, modulation yields the interpretation that *Don Quixote* is a novel, and so it refers to both possibilities to which the conclusion refers, and thereby transforms the inference into a necessary one. And the participants tended to accept it. The same contrast occurred with inferences from conditionals (Orenes & Johnson-Laird, 2012). A recent study examined four sorts of inference, and showed that participants accepted inferences that are necessary, and rejected those that are not necessary, regardless of whether or not the inferences are valid (Ragni & Johnson-Laird, 2020).

Conditionals also refer to conjunctions of possibilities that hold in default of knowledge to the contrary. Barrouillet and his colleagues asked children to list the possibilities for a conditional rule, such as:

- *If you wear a white shirt then you wear green trousers.*  [If A then B]

Third grade 8- to 9-year-old children tended to list just one possibility:

- *You wear a white shirt and you wear green trousers.*  1. [A & B]

Sixth grade 11- to 12-year-old children tended to list an additional possibility:

- *You don’t wear a white shirt and you don’t wear green trousers.*  2. [Not-A & not-B]

Only 14- to 15-year-olds in ninth grade, and adults, tended to list all three possibilities, including:

- *You don’t wear a white shirt and you wear green trousers.*  3. [Not-A & B]

The better predictor of how many of the three possibilities individuals list is, not their age, but the processing capacity of their working memory (see Barrouillet & Lecas, 1998; 1999; Barrouillet et al., 2000). All three inferences are necessary, but none are logically valid. For example, consider a case in which you don’t wear a white shirt. In standard logic, that suffices for its doppelganger for a conditional to be true, but of course the conjunction in the first of the conclusions above is false, which is a counterexample to the inference.

Many of the preceding inferences that participants tended to reject were alethically possible, but they had no way to register this assessment. Inferences that are not possible have conclusions that are inconsistent with the premises.

## Presupposed possibilities and conditionals

The model theory postulates that the possibility of an event presupposes the possibility that it does not occur. To assert, say:

*Trump may win*,

is to presuppose:

*Trump may not win*,

and vice versa. Participants tended to accept inferences of these sorts (Ragni & Johnson-Laird, 2020). Suppose the presupposition that Trump may not win is, in fact, false, then it is not the case that Trump may not win. It follows that Trump is certain to win, and this claim overrules both the preceding assertions. Following Strawson (1950), the model theory therefore assumes that an assertion’s presuppositions also hold for its negation. The mental representation of a possibility and its presupposition calls for an intuitive model of the possibility and a deliberative model of its presupposition in which it does not occur. Readers might suppose that *possibly A* and *possibly not A* also mutually imply one another in standard modal logics. They do not. The reason is that in all modal logics (except the simplest one, system K), the categorical assertion of *A* implies *possibly A*, and so if the latter implies *possibly not A*, it follows that that *A implies possibly not A*. No modal logic has this implication, because it would lead to absurdities, such as:

- *Necessarily it is snowing*.
- $\therefore$ *Possibly it is snowing*.
- $\therefore$ *Possibly it is not snowing*.

The conclusion contradicts the premise.

Conditionals have *if*-clauses that are subordinate to their main *then*-clauses, and that refer to possible situations that therefore presuppose that the situations may not occur. They hold in default of knowledge to the contrary. For instance:

*If there’s a triangle then there’s a star*

refers to the possibility that there’s a triangle, in which case there is a star. But, the possibility of a triangle presupposes the possibility that there is not a triangle. The conditional therefore has these two intuitive models:
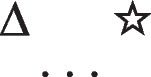


The first model represents the possibility of a triangle and a star, and the second model, the ellipsis, is a place holder for the presupposition, which allows that there’s a star and that there isn’t a star. These possibilities are made explicit in the deliberative model of the conditional, which includes the two possibilities given the presupposition, which the diagram demarcates with brackets:
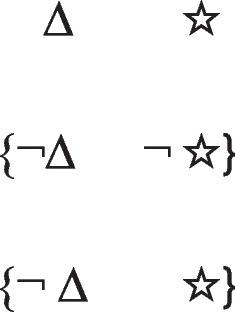


The negation of the preceding conditional can be expressed as:

*If there’s a triangle then there is not a star:*

And it has these deliberative models, because the conditional’s presupposition holds for its negation:
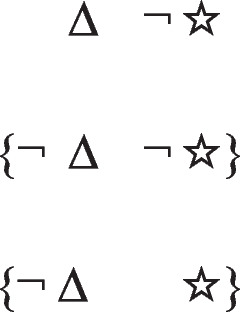


Because presuppositions hold for both the affirmation and the denial of conditionals, they have several crucial consequences.

The first consequence is that presuppositions play no role in deciding whether a conditional is true or false, because they hold in both cases. So, as a consequence, only two sorts of observation are relevant to a conditional’s truth value. It is true in case both its clauses are true, and no case in which its *if*-clause is true and its *then*-clause is false. The affirmative conditional above is therefore true given an occurrence of a triangle and a star and no occurrence of a triangle without a star. Likewise, the negative conditional above is true given an occurrence of a triangle without a star and no occurrence of a triangle with a star. A reviewer doubted the psychological plausibility of this account, on the grounds that it calls for a complex deliberation to grasp the cases hold for both the affirmation and the denial of a conditional. We agree that such a deliberation would be implausible. However, individuals do not even need to realize that *if*-clauses have presuppositions. All they have to do to verify a conditional is not to consider evidence in which its *if*-clause is false, which is easy because it doesn’t even match one of its intuitive models. And that’s what they do (for a review, see Schroyens, 2010). But, if they have to enumerate what is possible according to a conditional, they deliberate about each case in turn, and so they include those cases in which both clauses of a conditional are true and the two cases corresponding to its presuppositions (Goodwin & Johnson-Laird, 2018). These cases are possible, but they cannot verify the conditional, because they are also possible for false conditionals. Of course, the reviewer is right that there could be a better theory (cf. Barrouillet, Gauffroy, & Lecas, 2008). But, the model theory’s account is corroborated in studies of verification – people often treat the cases corresponding to presuppositions as “irrelevant” (e.g., Evans, 1972; Johnson-Laird & Tagart, 1969; Schroyens, 2010).

A second consequence of a conditional’s presuppositions is to solve the “paradox” of confirmation that occurs given a standard logic (Hempel, 1945). It occurs because a conditional such as:

*If an object is a black hole then it has a massive gravity*

is equivalent in any standard logic to:

*If an object doesn’t have a massive gravity then it’s not a black hole.*

The observation of a teddy bear supports this second conditional: the teddy doesn’t have a massive gravity and it isn’t a black hole. It therefore also supports the equivalent first conditional. That the observation of a teddy bear is even pertinent to the gravitation of a black hole is preposterous. In the model theory, however, the two conditionals are not equivalent. The confirmation of the first conditional demands the observation of at least one black hole with a massive gravity, and no observations of them that do not have a massive gravity. Teddy bears are impertinent.

Probabilists explain the paradox of confirmation too. They argue that the probability of a conditional equals the conditional probability of its *then*-clause given its *if*-clause. So, the conditional about the black hole and its converse differ, because their probabilities can differ. But why should the probability of a conditional equal the corresponding conditional probability?

The third consequence of a conditional’s presuppositions answers this question. They are irrelevant to its probability, because they also hold for the negation of the conditional (Khemlani et al., 2015a, 2015b). So, the probability of a conditional is equal to the conditional probability of the *then*-clause given the *if*-clause – a hitherto unexplained claim of probabilistic theories of conditionals (see Lopéz-Astorga, Ragni, & Johnson-Laird, 2022).

Any assertion about a possibility presupposes the possibility of its non-occurrence. It has an intuitive model of its occurrence, and a deliberative model of its non-occurrence. A striking prediction is therefore that those individuals who consider only the intuitive model will make an erroneous interpretation that the situation to which the assertion refers is one that occurred in reality, because a single model of a possibility corresponds to a factual claim. Only those individuals who consider both models – the intuitive one of the occurrence and the deliberative one of the non-occurrence – will make the correct interpretation that the situation is no more than a possibility. A recent study (Ragni & Johnson-Laird, 2020, Experiment 4) corroborated this prediction. It called for participants to draw their own conclusions from premises of this sort:

- *It is possible that Alex is in Erie.*  [Possibly A]
- *If Alex is in Erie than Eddy is in Fremont.*  [If A then B]

What, if anything, follows?

The intuitive model of the premises is:

- Alex in Erie      Eddy in Fremont

where, as earlier, we use words in a diagram of a model rather than a depiction. Those participants who consider only the intuitive model will tend to draw the erroneous categorical conclusion:

- $\therefore$ Eddy is in Fremont.  [$\therefore$ B]

But those participants who deliberate can construct the following models of the two possibilities in which Alex is not in Erie:

- {¬Alex in Erie  ¬ Eddy in Fremont}
- {¬Alex in Erie     Eddy in Fremont}

It follows from the intuitive and deliberative models that the necessary conclusion is:

- $\therefore$ *It is possible that Eddy is in Fremont.*  [$\therefore$  Possibly B]

Almost all the participants spontaneously formulated conclusions that were categorical and wrong, or modal and correct, with a slight bias towards the erroneous categorical ones. Analogous results occurred with other sorts of premises in the study, including those with conditionals that were asserted only as possible:

- *Alex is in Erie.*
- *It is possible that if Alex is in Erie then Eddy is in Fremont.*
- What follows?

The participants again tended to draw either the categorical conclusion or one asserting the possibility that Eddy is in Fremont, in roughly equal proportions.

Assertions in the subjunctive mood are a particular challenge to standard logics. The following vignette illustrates some of the difficulties:

He didn’t go to university. If he had, he would have studied hard. And he would have majored in economics. Either he would found it engaging or he might have left university. If he had left university, he would have worked as a librarian. And then if he had succeeded in getting his first novel published, he would have become a full-time novelist as happened in reality.

In many Indo-European languages, the *if*-clauses of conditionals can have a special mood – signaled in English by the past tense of the auxiliary verb, “have” – that sets up an alternative situation to the one currently holding in the discourse. The *if*-clause of the first conditional in the vignette sets up a counterfactual situation in which he went to university. Theorists sometimes describe counterfactuals as though they occur only with conditionals. They do not. After the first conditional in the vignette, there is assertion of a single counterfactual clause that he would have majored in economics. Next there is a counterfactual disjunction. The counterfactual conditional that follows creates an alternative to the current counterfactual situation: the protagonist now works in a library. The final counterfactual conditional creates yet another situation in which he gets a novel published, but this situation holds in reality as at the start of the vignette. The subjunctive mood can refer to an alternative to the current situation, and so its use can create a succession of alternatives to alternatives. Once an alternative is set up, the use of subjunctives to refer to them is not limited to conditionals, subjunctive assertions of any sort can do so too.

A seminal argument due to Adams (1970) draws a sharp distinction between factual conditionals in the indicative mood and counterfactual conditionals in the subjunctive mood. He drew the following contrast. This factual conditional is true:

*If Oswald didn’t shoot Kennedy then someone else did*.

But, the seemingly parallel counterfactual conditional could be false:

*If Oswald hadn’t shot Kennedy then someone else would have.*

So, the two sorts of conditional differ in a radical way. The contrast led Lewis (1973) to use the semantics of the standard sentential calculus for indicative conditionals, but to use a semantics of possible worlds for counterfactual conditionals. However, Adams’s argument contains a confound (Byrne & Johnson-Laird, 2019). Everyone knows that someone shot Kennedy, so the indicative conditional above must be true, whereas this knowledge has no bearing on the truth or falsity of the counterfactual conditional. The radical difference is therefore in background knowledge. A genuine parallel with the counterfactual conditional is instead an indicative conditional for which the fact that someone shot Kennedy is irrelevant, for example:

*If Oswald hasn’t shot Kennedy then someone else will.*

Prior to the assassination, the truth of this factual conditional is just as debatable as that of the counterfactual. So, the mental models of counterfactual conditionals do run in parallel with those for factual conditionals (Byrne, 2005; Byrne & Johnson-Laird, 2019; Espino, Byrne, & Johnson-Laird, 2020; Quelhas, Rasga, & Johnson-Laird, 2018). Their principal differences are in the epistemic status of their models, and in the split they make between intuitive and deliberative models. Intuitive models of counterfactuals tend to represent both what is true – the negation of both its clauses, and what is false the affirmation of both clauses. So, the intuitive models of a counterfactual such as:

*If there had been a triangle then there would have been a star*

are shown here with their epistemic status:
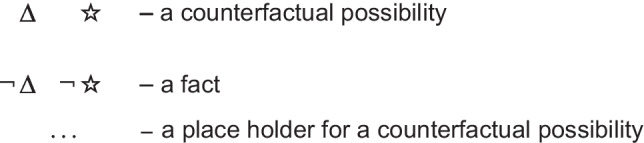


Byrne and her colleagues have corroborated this account: if an inference depends on the denial of a conditional’s *then*-clause, then individuals are more likely to make it from a counterfactual conditional than from a factual conditional (see, e.g., Byrne, 2017; Byrne & Tasso, 1999; Eichhorn, Kern-Isberner, & Ragni, 2018).

Another series of experiments examined participants’ preferred paraphrases of indicative and subjunctive conditionals with the same contents referring to events in the past, present, or future (Espino et al., 2020). Their preference was for paraphrases capturing the model theory’s semantics. For instance, an indicative conditional had the following favored paraphrase (translated from the Spanish, the language of the experiment) based on epistemic possibilities:

- *If he is injured tomorrow, then he will take some leave.*
- Paraphrase: *It is possible, and remains so, that he is injured tomorrow, and in that case certain that he would take some leave.*

Likewise, the preferred paraphrase of the corresponding counterfactual was:

- *It was once possible, but does not remain so, that he will be injured tomorrow*, *and in that case certain that he takes some leave.*

The paraphrases cannot be made in standard modal logics or in conditional logics, which lack a language combining time, possibility, and certainty.

Modal reasoning can also hinge on quantifiers, such as “all” and “some”. Aristotle analyzed such reasoning in syllogisms (see his *Prior Analytics*, Book 1, Barnes, 1984, Vol. 1; and Malink, 2014, for corrections and clarifications). A syllogism has two premises, and one of them contain a single quantifier, and the other premise either refers to a particular individual or else also has a single quantifier. We present examples of both sorts below. Each premise has only one intuitive model of the set of entities to which it refers. In the past, the model theory has been applied to quantified reasoning without modals, and to modal reasoning without quantifiers, but we unite these precursors here.

A quantified assertion such as: *All those architects are cyclists* has a single model representing the relation between the relevant set of individuals who are architects and the set of cyclists. The models vary in the number of individuals that they represent, though it tends to be small, and in how typical they are for the quantified premise, as observed in external models that reasoners constructed from cut-out shapes representing different sorts of entity (Bucciarelli & Johnson-Laird, 1999). These variations characterize differences in inferences from one individual to another, and within individuals from one situation to another – with the consequence that people differ vastly in the accuracy of their syllogistic reasoning (Khemlani & Johnson-Laird, 2022). The representation of possibilities follows the same principle as before: intuitive models represent the occurrence of the possibility, and deliberative models represent the presupposition that it did not occur. Here is an example:

- *All those architects are cyclists.*
- *Manuela may be one of those architects.*

They have a single intuitive model of the possibility to which they refer, and so each line represents a separate individual:
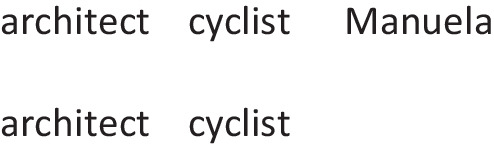


The model represents two architects, who are both cyclists, and one of them is Manuela. It is iconic in its use of a set of tokens to represent a set of individuals. And it represents Manuela as a member of the set of those architects who are cyclists. It predicts that those participants who consider only the intuitive model should err in drawing a categorical conclusion:

$\therefore$ *Manuela is a cyclist*.

A deliberative model represents the presupposition that the possibility did not occur, i.e., Manuela is not one of those architects (who are cyclists):

- ¬ architect ¬ cyclist Manuela
-    architect   cyclist
-    architect  cyclist

Deliberation takes into account the intuitive model too, and so participants who deliberate should draw the necessary modal conclusion:

- $\therefore$  *Manuela may be a cyclist*.

The two predicted conclusions, categorical and modal, were the most frequent in recent study in which participants drew their own conclusions (Quelhas, Rasga, & Johnson-Laird, 2023, Experiment 2).

## Condensation of consistent possibilities

Mental models need to be parsimonious given the limited processing capacity of working memory, and so one way to ease the load is to condense possibilities. The model theory postulates that two or more models of consistent possibilities can be condensed into a single model of one possibility in default of knowledge to the contrary. We define condensation as follows:

Given any number of individual possibilities, such as *possibly A* and *possibly B*, their condensation occurs in default of knowledge to the contrary and yields a conjunction: *possibly (A and B).*

Experiments have shown that participants do tend to condense separate but consistent possibilities into one (Ragni & Johnson-Laird, 2020), for example:

-      *It is possible that Ann is in Bath and it is possible that Ben is in Ayr.*
- $\therefore$  *It is possible that Ann is in Bath and that Ben is in Ayr.*

But they reject such condensations when the two possibilities are inconsistent, as in:

*It is possible that Ann is Bath and it is possible that Ann is in Ayr*.

Condensation explains certain systematic inferences of *or*-deletions, which are inferences from a disjunction to one of its clauses, or even to a conjunction of both of them. We have already illustrated examples of such *or*-deletions (due to Hinterecker et al., 2016), for example:

-      *There is a square or a there is a circle, or both*.
- $\therefore$  *Possibly, there is a square*.

A well-known “paradox of free choice permission” is similar except that it depends on an explicit disjunction of deontic alternatives concerning what is permissible (Kamp, 1973), for example:

- *     You are allowed to smoke or to drink.*
- $\therefore$  *You are allowed to smoke.*

Such an inference is a flagrant violation of the standard meaning of “or”, which refers to alternatives, as in: *She had a smoke or she had a drink, or both* (see Appendix 1). So, hundreds of articles have sought to explain the phenomenon, using either pragmatics or semantics. The starting point of pragmatic explanations is Grice (1989), and there are Gricean, neo-Gricean, and post-Gricean hypotheses (e.g., Bar-Lev & Fox, 2020; Kratzer & Shimoyama, 2002; Tieu et al., 2016). The semantic accounts tend to treat one interpretation of disjunctions as lists (e.g., Zimmerman, 2000; Geurts, 2005; for a review of both approaches, see Johnson-Laird, Quelhas, & Rasga, 2021).

In fact, the model theory has a pre-hoc explanation of free choice permissions and other *or*-deletions (Johnson-Laird et al., 2021). It predicts the paradox on the grounds that a disjunction of explicit deontic possibilities, i.e., what is permissible, yields a conjunction of deontic possibilities. The parser substitutes them for the epistemic possibilities that otherwise occur in the interpretation of disjunctions of categoricals. Gricean theories concern single utterances (Cohen, 1971; see also Bar-Lev & Fox, 2020), and so they cannot explain the following inference, which depends on three assertions:

- Your professor told you that you are permitted to do only one of the following actions:
  -     *You can do your homework.*
  -     *You can do the presentation slides.*
  - $\therefore$ *You are permitted to do your homework.*

Participants were happy to accept such an inference (Rasga, Quelhas, & Johnson-Laird, 2022). Likewise, an explicit disjunction of epistemic possibilities also yields *or*-deletions as in:

- *     It is possible that the fire burnt down the yellow house or that it burnt down the green house, but not both.*
- $\therefore$ *The fire may have burnt down the yellow house.*

Most participants accepted such inferences (Rasga et al., 2022).

The most striking *or*-deletions are those to which the model theory led. They yield categorical conclusions rather than modal ones. They occur as a result of the condensation of the epistemic possibilities to which they refer. And the condensation depends on knowledge that the disjunction is not primordial: it does not refer to a set of exhaustive alternatives for a single situation (see the section on *the model theory of modals*), for example:

-      *She is taller than the boy or the man.*
- $\therefore$ *She is taller than the boy.*

The *or*-deletion is just one interpretation of the disjunction, but participants tended to accept the inference (Rasga et al., 2022). A primordial interpretation is also available, as in:

*She is taller than the boy or the man, but not both.*

It blocks the *or*-deletion.

## Local consequences of inconsistency

If a set of assertions yields a conjunction of two sets of models that are inconsistent with one another, then it returns the empty model. Its symbol, nil, denotes that there are no possibilities to which the assertions refer (see Appendix 2). The conjunction of the null model with any other model yields only the null model again. So, inconsistencies have only local consequences: something is wrong with the assertions on which they are based. When participants are given the following premises, for example:

- *If a pilot falls from a plane then the pilot dies.*
- *This pilot fell from a plane but did not die.*

they notice the inconsistency, but it does not inspire them to draw arbitrary conclusions (Johnson-Laird et al., 2004). Instead they search for an explanatory resolution. They prefer, not a minimal revision of the premises, but a causal explanation, as in the following examples:

- *The plane was on the ground and so he didn’t fall far*.
- *The pilot fell into a deep snow drift.*
- *The pilot was already dead.*

## Summary

Table 1 summarizes the four principles of the model theory, and presents examples from some of the experiments corroborating them with citations to the relevant articles. The evidence in this section bears out the model theory’s essential principles, and their predictions about likely errors and necessary conclusions.

**Table 1:** The model theory’s four essential principles for modal reasoning and examples of their experimental corroborations with their citations

**1. Alethic necessity of correct inferences** Necessary inferences are those with conclusions that refer only to possibilities or facts to which the premises refer, and do not deny any of them.	
Participants accept inferences that are necessary but not valid, e.g.: *It’s cloudy or it’s windy.* $\therefore$ *It may be cloudy.*	Hinterecker et al., 2016
Participants reject inferences that are not necessary but valid: *Possibly it’s cloudy or else it’s windy, but not both.* $\therefore$ *Possibly it’s* c*loudy or it’s windy, or both.*	Ragni & Johnson-Laird, 2020
**2. Presupposed possibilities** The possibility of a situation (represented in an intuitive model) presupposes the possibility that it does not occur (represented in a deliberative model).	
Participants accept this inference and its converse (both invalid): *Possibly it’s raining.* ∴ *Possibly it’s not raining.*	Ragni & Johnson-Laird, 2020
Given: *If it’s cloudy then it’s windy*, participants choose this paraphrase: *It’s possible, and remains so, that it’s cloudy, and in that case* *certain that it’s windy.*	Espino et al., 2020
Participants draw their own conclusions of this sort: *All those men may be tall. Bud is one of those men.* $\therefore$ *Bud may be tall.* A common error is: $\therefore$ *Bud is tall.*	Quelhas et al, 2023
**3. Condensation of consistent possibilities** If modulation—the use of meaning and knowledge—assesses possibilities as consistent then their models are condensed into one model of a possibility.	
People modulate: *He is at home or at the office,* to yield an exclusive interpretation. Given: *He is at home*, they infer: $\therefore$ *He is not at the office*.	Quelhas & Johnson-Laird, 2017
Participants accept the necessary inference, which is invalid: *Possibly it’s cloudy and possibly it’s windy.* $\therefore$ *Possibly it’s cloudy and windy*.	Ragni & Johnson-Laird, 2020
Participants accept the necessary but invalid inferences: *You can have steak or sole.* $\therefore$ *You can have steak.* (A paradox of free choice permission.) *Some customers ate steak or sole.* $\therefore$ *Some customers ate steak.*	Rasga et al., 2022
**4. Local consequences of inconsistency** Inconsistencies yield the null model (of no possibilities), which have only local consequences.	
Participants try to explain the origin of an inconsistency: *If a pilot falls from a plane then the pilot dies.* *This pilot fell from a plane but did not die.* Participants suggest possible explanations, such as: *The pilot was already dead.*	Johnson-Laird et al., 2004

## Is a modal logic a feasible basis for human reasoning?

A potential alternative to the model theory of modal reasoning is a cognitive theory relying on a modal logic. In the *Introduction*, we cited the first such theory due to Osherson (1976) and its less than convincing results. So, the first part of this section assesses psychological studies of modal logics’ semantic theory of possible worlds as an alternative to the model theory, and the second part considers the feasibility of logic-based theory in the light of our experimental results.

## Psychological studies of modal logic and possible worlds

As far as we know, no crucial experiment has yielded evidence about modal reasoning contrary to the model theory and in support of an alternative theory. But current critiques report results supporting a possible-worlds semantics. Perhaps what prepared the ground for these studies was Kripke’s (1980) reinterpretation of possible worlds as miniworlds rather than the vast worlds that Leibniz envisaged (see Appendix 1). So, we now consider the psychological evidence for possible worlds.

The conditional logics of Stalnaker (1968) and Lewis (1973) led Rips and Marcus (1977) to argue for a version of such a semantics. It defines the situations in which conditionals are true, though Lewis accepted Adams’s (1970) confounded argument for a radical difference between factual and counterfactual conditionals (see the earlier subsection on *presupposed possibilities and conditionals*). A more recent study applied possible worlds to reasoning problems in the Graduate Record Examinations (GRE), which are informal and often concern possibilities (Yang, Bringsjord, & Bello, 2006). Their theory used both formal rules and possible-worlds, but the authors pointed out that the model theory is a special case of their theory.

A well-known result in the reasoning literature is Byrne’s demonstration that an additional premise can suppress a valid – and necessary – inference (see Byrne, 1989, 1991;

Espino & Byrne, 2020). From premises such as:

- *If she meets her friend then she will go to a play.*
- *She met her friend.*

participants inferred:

∴ *She went to a play*.

But, with the additional premise:

- *If she meets her friend then she will go to a play.*
- *If she has enough money then she will go to a play.*
- *She met her friend.*

participants tended not to draw the conclusion. Modulation can lead to a condensation that predicts this performance. Individuals condense the possibilities referred to in the two *if*-clauses into a single model (*meeting her friend* and *having enough money*), so that both are required for the event in the *then*-clause to occur (*going to a play*). Because suppressions are contrary to standard logics, many counter-proposals have been made about them, for example, Cariani and Rips (2017). So far, however, they have not led to experimental results contrary to the model theory.

In a different sort of study, De Brigard and his colleagues instructed participants to read fictional descriptions of impossible events, such as, “a kitten that hatches from an egg”. The participants next had to imagine a “possible world” in which the description was true, and then to rate on a 9-point scale the similarity of this situation to the actual world (De Brigard, Henne, & Stanley, 2021). They also had to rate the plausibility of the counterfactual description on the same scale. The two ratings were highly correlated, and analogous results occurred when episodic memories concerned counterfactual events (Stanley, Stewart, & Brigard, 2017). These findings reveal the participants’ creativity, but they seem to be neutral between possible miniworlds, mental models, or some other way to represent possibilities.

Skovgaard-Olsen, Collins, and Klauer (2023) carried out an ingenious study based on possible worlds in non-standard theories of conditionals (Stalnaker, 1968; Lewis, 1973), and they argued that the model theory cannot explain their results. In their main experiment, most participants judged a conditional to be true when both its clauses were true in a photo that served as evidence, and most of them judged a conditional to be false when its *if*-clause was true in the photo but its *then*-clause was false. These two results are almost impervious to experimental manipulations (see Schroyens’ (2010) meta-analysis of verifications of conditionals). Unusual results occurred in the experiment when conditionals had an *if*-clause that was false in the photo. For instance, participants read:

*If this is one of Jack’s photos of the railroad station, then there is a fruit bowl in it.*

and they saw a photograph of a kitchen containing a fruit bowl. They knew that the only alternative photo to the one they were looking at was of a railroad station with a warning sign about the platform’s edge, but no fruit bowl. They had to judge whether the conditional was *true*, *false*, or *neither true nor false*, about the photograph. Its *if*-clause was false because the photo was of the kitchen, but its *then*-clause was true because the photo contained a fruit bowl. Nearly half of the participants judged the conditional to be false, and most of the rest of them judged it to be true. A similar distribution of responses occurred when both clauses of a different indicative conditional were false in the photo. When the *if*-clause of a third conditional was false, but its *then*-clause referred to an object that was in neither photo, most participants evaluated the conditional as false. The experiment also tested counterfactual conditionals in the same ways. The investigators interpreted their results for conditionals with false *if*-clauses as contrary to the model theory.

The model theory treats evidence in which a conditional’s *if*-clause is false as consistent with the truth or falsity of the conditional (see the subsection above on *presupposed possibilities and conditionals*). Such evidence has no bearing on the truth value of the conditional, and experiments have corroborated this account – it tends to be judged as “irrelevant” (e.g., Evans, 1972; Johnson-Laird & Tagart, 1969; see also Goodwin & Johnson-Laird, 2018). Schroyens’ (2010) meta-analysis revealed variations in judgments of the truth values of conditionals with false *if*-clauses. And he correctly pointed out that as a given category of judgment “irrelevant” can be a misleading option for conditionals with a false *if*-clause. Instead, as he remarked, they are consistent with its truth or falsity. Gauffroy and Barrouillet (2014) used the option “one cannot know” in a verification study, and their participants selected it when the evidence showed that both clauses of a conditional were false. The conclusion of Schroyens’ (2010, p. 917) meta-analysis was that the model theory can explain all the basic phenomena of the verification task, but that the options for participants to record their judgments are crucial, and so too is whether the evidence for the falsity of an *if*-clause depends on its explicit denial or, as in Skovgaard-Olsen and his colleagues’ experiment, an implicit one. Contrary to their claim, however, the model theory can explain their results.

The split between judgments of “true” and “false” for conditionals with false *if*-clauses signals uncertainty within or between participants. Three factors are pertinent. First, no option allowed the participants to respond that such conditionals could be true or false. Second, modulation played a major part in the experiment: the participants knew that each photo was one of a disparate pair. They also knew when an entity was not in either photo in a pair, leading them to select “false” as their evaluation. Third, the design held the two pieces of evidence – the photos – constant, and manipulated the contents of the conditionals to create five sorts of trial for each pair of photos. One consequence of these factors is that the parallel between conditionals with factual and counterfactual interpretations no longer holds for those with false *if*-clauses (pace Skovgaard-Olsen et al., 2023, p. 19). For instance, given the counterfactual:

- *If this had been one of Jack’s pictures of a railroad station, there would have been a warning sign about standing too close to the edge in it*

participants looking at the photo of the kitchen should be biased towards judging it true, because it *is* a true counterfactual. But, given the indicative conditional and the same photo:

- *If this is one of Jack’s pictures of a railroad station, then there is a warning sign about standing too close to the edge in it*

the participants noticed the conditional does not match the photo, and were biased to judge it false. In sum, science would hardly be feasible in a comparable case in which evidence that one of Jupiter’s moons has weak gravity falsified the hypothesis:

*If this object is a black hole then its gravity is vast*.

Our earlier evidence corroborated that conditionals presuppose possibilities corresponding to the falsity of their *if*-clauses. And it also supports the model theory’s explanation of the present experiment. Not every result in a verification task is a consequence of the meaning of the sentence to be verified (Johnson-Laird, Byrne, & Khemlani, 2023). So, none of the studies of possible worlds casts robust doubt on the model theory.

## Towards a cognitive theory embodying modal logic

A cognitive theory of modal inferences based on an appropriate logic might lead to a better understanding of human reasoning. It might also improve the model theory and even suggest a new way to think about logic. But, before we make some suggestions of this sort, we need to point out a methodological difference between the two domains. Logicians call for “formally precise” definitions, and a like-minded reviewer requested a formal specification of the model theory. Cognitive psychologists do not tend to formulate theories in this way. They aim for a theory that is clear enough both for empirical tests and for computer simulation. One advantage of computer implementations over formalizations is illustrated in Wolfgang Schwarz’s program for assessing putative theorems in standard sentential and predicate calculi and their modal counterparts (https://www.umsu.de/trees/). It is much easier to use the program than to have try to find a proof by hand. Another advantage is that the process of programming can lead to new ideas, for example, the discovery of illusory inferences.

A sensible starting point for intrepid “cognitivists” aiming to construct a logic-based theory of human reasoning is the model theory. The present section therefore begins with its theoretical implications, and its four essentials for modal reasoning.

Views about errors in reasoning have swung from one extreme to another. Not so long ago, theorists believed that it was impossible for humans to err in logical reasoning (e.g., Henle, 1962; Smedslund, 1970) or that their errors were unsystematic (e.g., Rips, 1994). But, illusory inferences are systematic and have no plausible basis in anything other than the process of reasoning (Khemlani & Johnson-Laird, 2017). Systematic errors, such as inferences of categorical conclusions from descriptions of a possibility, are not easy to accommodate within the formal rules of inference in a modal logic. Rules that generate such errors would be inconsistent with correct rules, and the mixture would be a recipe for disaster. Pragmatics might help, but again with the risk of inconsistency. A realistic alternative may be to forget about errors, and to use logic solely as a guide to correct modal inferences. This goal also avoids the problem of explaining which particular conclusions human reasoners tend to draw from given premises, and why they decide that “nothing follows” from certain premises. Both are impossible to do in standard modal logics, because an infinity of different conclusions follows validly from any premises (see the earlier section on *the original theory of mental models*).

Humans reason from premises stated in the vernacular; but logics use formal languages. So, a cognitive system has to translate everyday language into the logical forms that match those of axioms and rules of inference. No algorithm exists to carry out this translation, and it is surely one reason for Bar-Hillel’s lament in an epigraph to this paper. The difficulties that arise are exemplified in Keene’s (1992) translations of real arguments into sentential or predicate logics. The fundamental problem is that logical form cannot be retrieved from syntax alone. For instance, the following sentence:

*He likes eggs and he likes bacon but he doesn’t like eggs and bacon*

could be assigned the self-contradictory form:

(E & B) & not (E & B).

But it need not be a self-contradiction, because the “and” in “eggs and bacon” is a synonym for “together with”. So, a translation into logical form depends on meaning and knowledge.

Earlier we described four essentials of standard modal logics: their criterion of correct reasoning is validity, the inference from a categorical assertion to its possibility is valid, the condensation of consistent possibilities is invalid, and inconsistent premises yield a valid inference of any conclusion whatsoever. The model theory rejects all of them. And its alternative essentials for modal reasoning are corroborated in the results summarized in Table 1 above. So, what follows are suggestions for how the cognitivists might deal with them in devising their theory. They be could the starting point of an adversarial collaboration.

1. Reasoners accept necessary inferences even if they are logically invalid, such as:

- *If Evelyn visited Rome then she visited Naples.*
- $\therefore$ *Possibly Evelyn visited Rome and Naples.*

Such inferences are so convincing that no-one realized until very recently that they are not valid, for example, a counterexample in a standard logic is that it is false that Evelyn visited Rome, and so the conditional premise is true, but the conclusion is false. The conditional logics of Stalnaker (1968) and Lewis (1973) are major improvements over standard modal logics for the cognitivists, but even they reject the preceding inferences. As Stalnaker wrote:

my theory invalidates all of these inferences, including the first (from If A, then B to the possibility of A and B). (p.c. to J-L, 10-24-2023)

What allows the inferences in the model theory is that conditionals assert possibilities in default of knowledge to the contrary. In addition, reasoners reject inferences that are not necessary even if they are valid. For example, they reject an inference from an exclusive disjunction to an inclusive disjunction:

- *It is possible that Ann is in Bath or it is possible that Tom is in Ayr, but not both.*
- $\therefore$ *It is possible that Ann is in Bath or it is possible that Tom is in Ayr, or both.*

It is not a necessary inference, because the premise does not refer to the possibility of both clauses holding, but the conclusion does. Yet, it is a valid inference in all standard modal logics. And reasoners are likely to reject an inference such as the following one, which is also not necessary but valid in all standard modal logics:

- If it rains then we may get wet.
- $\therefore$ If we bring umbrellas and it rains then we may get wet.

One solution is for the cognitivists to adopt alethic necessity as the criterion of correct inferences. It need not replace validity: both principles can be used to assess inferences, and they might lead to the discovery of a so far elusive rebuttal of the model theory – an acceptable inference that is valid but not necessary.

2. Reasoners treat the assertion of a possibility as presupposing the possibility of its non-occurrence. This assumption, which goes back to Aristotle (see the *Introduction*), is contrary to all standard modal logics that allow that the truth of an assertion implies its possibility, i.e., system T and the infinitely many logics embodying it (see Appendix 1). The incompatibility of the model theory’s presuppositions for possibilities and standard modal logics is at the heart of the differences between them.

3. Reasoners tend to condense consistent possibilities into one. As Hamlet famously remarked:

… *for there is nothing either good or bad, but thinking made it so*.

He meant that both the good and the bad result from thought. Such an inference is invalid in all standard logics. Yet, condensation led to the discovery of a new sort of *or*-deletion from categorical disjunctions. If modulation conveys that the two clauses in a categorical disjunction can be condensed into one, then reasoners will accept an *or*-deletion (Johnson-Laird et al., 2021; Rasga et al., 2022). A minimal illustration contrasts two inferences. Participants accepted inferences such as:

- *Eric drinks red or white wine at lunch.*
- $\therefore$ *He drinks red wine at lunch.*

But they rejected:

- *Eric drank red or white wine at lunch today.*
- $\therefore$ *He drank red wine at lunch today.*

The importance of consistent possibilities in these *or*-deletions is illustrated in this pair of contrasting inferences from another experiment (Johnson-Laird et al., 2021, Experiment 4). Participants tended to accept the inference:

- *Some of the students chose acting or dancing.*
- $\therefore$ *Some of the students chose acting.*

but to reject the inference:

*All of the students chose acting or dancing.*

$\therefore$ *All of the students chose acting.*

The two proportions of students can be condensed into the same possibility in the first inference, but not in the second inference. Similar contrasts occurred between premises with these contrasting pairs of quantifiers: *one* versus *none*, and *few* versus *most*. None of these inferences is valid in standard logics. To accommodate them in the cognitivist theory calls either for a more powerful pragmatic account than exists at present, or the adoption of condensation. Many of the inferences that it produces are necessary in the model theory, and a defeasible modal logic could treat them as valid, too.

4. Reasoners treat inconsistencies as local: they search for an explanation to resolve them. In the model theory: an inconsistency yields the null model representing that the premises refer to no possibilities, and it creates only the null model again in conjunction with any other premises. Yet, inconsistencies are a disaster in standard modal logics, because valid inferences of any conclusions whatsoever follow from them. They can have no counterexamples. Cognitivists might be able to prevent the explosive consequences of inconsistencies by adopting a “paraconsistent” modal logic, which tolerates inconsistencies, and does not allow any conclusions whatsoever to be drawn from them (cf. Odintsov & Wansing, 2017; Priest, Tanaka, & Weber, 2022). A more modest proposal is to adjust the formal rules of a standard modal logic to block the inferential consequences of an inconsistency. Rips (1994) constrained a rule such as: *A*; $\therefore$ *A or B or both*, by restricting its use to finding a step in a proof that works backwards from a given conclusion. Other theorists adopted analogous constraints (e.g., Braine, 1978; Johnson-Laird, 1975). But, none of these restrictions prevent a proof of a *given* arbitrary conclusion, *B*, from inconsistent premises (see the proof that a rhino is in your bath in the section on *the four essentials of standard modal logics*). In comparison, an amendment to the definition of validity is simple: add a rider to its definition requiring the premises to be consistent; and so inconsistencies no longer yield any valid inferences.

Perhaps the greatest difficulty for cognitivists aiming to formulate a competence theory of modal reasoning is a fundamental difference between how individuals think of possibilities in daily life and how standard modal logics treat them. The model theory postulates that humans represent possibilities as modifications to models of reality. Consider this assertion:

The cricket ball would have hit the wicket if it had not hit the batsman.

According to the model theory, to evaluate this claim about a counterfactual possibility, you imagine the trajectory of the ball but remove the batsman from your kinematic simulation. Umpires in several sports make such simulations, and their judgments in cricket are quite accurate, as checked using the Hawk-Eye TV system, which also constructs the counterfactual trajectory. The use of such simulations has also been corroborated in experiments on evaluations of causal relations (Gerstenberg et al., 2021; Johnson-Laird, Byrne, & Khemlani, 2024; Khemlani, Goodwin, & Johnson-Laird, 2015).

The contrast with standard modal logics is apparent in inferences such as:

-      *Russell sat at the rear of the seaplane.*
-      *It is possible that all those at the rear of the seaplane escaped.*
- $\therefore$ *Russell may have escaped.*

Reasoners can build an intuitive and kinematic model of Russell sitting at the rear of the plane and everyone there escaping, and then perhaps they build deliberative model in which not everyone there escaped including Russell. The two models together yield the modal conclusion above. So, reasoners start with a model of reality: Russell sitting at the rear of the plane, and they add to this intuitive model that everyone there escaped. Their deliberative model represents the presupposition that not everyone at the rear of the plane escaped. Hence, Russell’s escape is only a possibility. In standard modal logics, the inference is invalid in a revealing way. It has a counterexample in which Russell is sitting at the rear of the plane in the real world, whereas those who escaped from the rear of the plane are in an alternative possible world. Why don’t any standard modal logics consider the possibility embodied in the intuitive model above? It is because modal logics operate on sentences, not models, and so they have no way to include Russell in the set of passengers at the rear of the plain when they examine inferences from the premise asserting the possibility of their escape.

Nothing in a standard modal logic can justify the withdrawal of a valid inference. To deny its conclusion creates an inconsistent set of assertions (for the awful consequences, see the earlier section on explosive inconsistencies). Given an inference, such as:

-       *There may be a ban on watering lawns or else there may be a drought.*
-      *There cannot be a ban of watering lawns.*
- $\therefore$ *There may be a drought.*

and the subsequent discovery that a drought is impossible, a sensible reaction is to withdraw the conclusion. But, no standard logic provides any justification for doing so. A sensible solution is therefore to adopt a defeasible modal logic – a “nonmonotonic” one in AI jargon (e.g., Areces et al., 2023; McDermott, 1982).

The formulation of a cognitive theory of modal reasoning based on logic will not be easy even with the restricted goal of accounting only for correct inferential competence. The simplest solution may be to convert the model theory insofar as possible into a modal logic (cf. Bringsjord & Govindarajulu, 2020). The project, though difficult, may be viable, and it may be worthwhile beyond a mere demonstration that standard modal logics can account for human inferential competence.

## Conclusions

The mental model theory makes corroborated predictions about modal reasoning. No alternative account exists as yet. And, as we argued at the outset, theories of probabilistic reasoning are unlikely to extend to modality: the meanings of permissibility and obligation cannot be captured as tacit assertions about probabilities. It remains an open question whether a cognitive theory assimilating a modal logic is feasible. It would demand major departures from standard and non-standard modal logics. A corollary is that the implementation of the model theory in the mind confers several advantages on human reasoning that are not available from either probabilistic or standard logics. It allows reasoners to draw their own conclusions or to decide that none is worth inferring. It allows them to withdraw conclusions, even those from necessary inferences. In logic, an inconsistency has catastrophic consequences. Anything goes. In Wittgenstein’s 1939 course on the foundations of mathematics he had discussions with Turing – perhaps the only conversations between the two of them of which there are records – and it seems to have perplexed them both that a small inconsistency has big consequences (Wittgenstein & Bosanquet, 1989, p. 211). Everyone is likely to hold inconsistent views – to check the consistency of, say, 20 beliefs can call for over a million assessments (i.e., 2^20^), i.e., the task is computationally intractable (and NP complete, see Cook, 1971). In the model theory, inconsistencies yield only the null model, which has only local consequences.

If the model theory is right, the inferential system constructs models of possibilities that each hold in default of knowledge to the contrary. They enable reasoners to make necessary inferences, which are those with conclusions that only refer to possibilities or facts to which the premises refer, and to balk at conclusions that introduce new possibilities even in inferences that preserve truth, because they are valid. Epistemic possibilities, which depend on empirical knowledge of the world, come in degrees (White, 1975; Lassiter, 2017; Johnson-Laird & Ragni, 2019), and therefore underlie conclusions of varying probabilities. They also depart from standard modal logics in that, as Aristotle intimated, a possible situation presupposes the possibility that it does not occur. The theory therefore differs in many ways from all standard modal logics, and so if one of them is to provide a normative account of correct reasoning it will need radical changes.

## Appendix 1. Modal logics and possible worlds

This appendix outlines modal logics and their semantics based on possible worlds (see, e.g., Chellas, 1980; Hughes & Cresswell, 1996). It considers two ways in which possible worlds can be tiny as opposed to vast, and therefore more compatible with cognition.

The sentential calculus for negation and connectives such as idealizations of “if” and “or” has a system of formal rules, which can be used to derive proofs. Various methods of proof exist, but nowadays the “tree” method is pre-eminent (see, e.g., Jeffrey, 1981, Ch. 2). Its typical form of proof is a “reductio ad absurdum,” in which the negation of the conclusion to be proved is added to the premises, and if the proof establishes that the resulting set of sentences is inconsistent – they cannot all be true, then the conclusion follows from the premises. And, to prove invalidity, it constructs a counterexample in which the premises are true, but the conclusion is false. Consider, for example, the application of the tree method to a sentential inference in English:

- *1. Ann is in Paris or Beth is in Rome, or both.*
- *2. Ann is not in Paris.*
- $\therefore$ *Beth is in Rome.*

where ‘$\therefore$’ stands for “therefore”. We replace the putative conclusion with its negation:

- *3. Beth is not in Rome.*

A formal rule of inference for disjunction allows us to add from the first premise its two alternative cases to the three numbered sentences above to form an inverted tree:
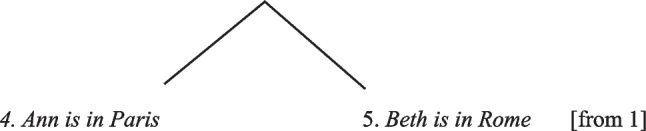


Sentence 4. is inconsistent with sentence 2; and sentence 5 is inconsistent with sentence 3. So, both cases yield inconsistencies. The first three numbered sentences are therefore inconsistent. If the denial of a conclusion is inconsistent with the premises, then the conclusion itself follows from the premises. So, the proof shows that the original conclusion follows from the premises.

Formal rules of inference allow manipulations of symbolic sentences without any need to consider their meanings. However, the sentential calculus has a semantics. It specifies that if a sentence is true then its negation is false, and vice versa. It also specifies that an inclusive disjunction is true, such as sentence 1 above, if and only if at least one of its two clauses is true, and so it is false only in case both its clauses are false. Such a semantics is known as “truth functional”, because the two potential truth values, *true* and *false*, for any well-formed sentence are a particular function of the truth values of its clauses. The semantics enables logicians to check that a proof corresponds to a valid inference. For instance, the consequences of the truth values of the premises in the proof above are as follows. Given that Ann is not in Paris (sentence 2), then the first clause in the disjunction (sentence 1) is false. On the assumption that the disjunction itself is true, its second clause must therefore be true, and so the inference to the conclusion: *Beth is in Rome*, is valid. Validity is the criterion for a correct inference in all standard logics, and an inference is valid provided that its conclusion is true in all interpretations of the premises according to their semantics (Jeffrey, 1981, p. 1). As we have shown, the inference has a formal proof, too. The sentential calculus is indeed *complete* in the sense that any valid inference according to its semantics is also provable in its formal system; it is also *sound* in the sense that any provable inference in its formal system is also valid in its semantics. Not all formal logics have these desirable properties. Indeed, Gödel’s “incompleteness” theorem, which we sketched in the introduction to this article, showed that there are truths in arithmetic that can be neither proved nor disproved in any consistent formal logic for the arithmetic of natural numbers.

If two modal operators, *possibly* and *necessarily*, are added to the sentential calculus together with their appropriate rules of grammar, inference, and semantics, they yield a modal logic. In most modal logics, the two modal operators are interdefinable (see the *Introduction* for the relevant definitions). So, strictly speaking, the logic needs just one of the operators, but it is convenient to have both.

The simplest modal logic is named system K, in honor of Kripke. It recognizes the truth conditions for possibly and necessarily, for example, *possibly it is raining* is true if *it is raining* is true in some relevant possible world, and *necessarily it is raining* is true if *it is raining* is true in all relevant possible worlds.

At this point, we can introduce Kripke’s (1963) striking innovation. He showed that the validity of inferences in modal logics that differ in their axioms (or, equivalently, their formal rules of inference) corresponds to a relation among possible worlds, which he referred to as “accessibility”, and which in effect is the relevance of one world to the truth values of sentences in another possible world. An example will clarify his insight. In system K, the following inference is unprovable:

-     *It is raining*
- $\therefore$ *Possibly, it is raining.*

The reason is that system K makes no assumptions about the accessibility (or relevance) of one world to another. However, the addition of the assumption that each possible world is accessible to itself – accessibility is a reflexive relation – creates system T, in which one can prove the inference above. The proof is as follows, where A denotes *it is raining*:

- 1. A in w                     (The premise holds in world real world, w)
- 2. ¬ possibly A in w    (The negation of the conclusion in w to be proved)
- 3. wRw                       (An axiom for the assumption that accessibility is reflexive)
- 4. ¬ A in w                 (An inference from 2 and 3, which holds in w)

Line 4 is inconsistent with line 1, and so the conclusion is proved:

- $\therefore$ possibly A in w.    ($\therefore$ *Possibly, it is raining.*)

The assumption that each world is accessible to itself underlies a countable infinity of distinct modal logics differing in which inferences are valid in them (Ragni & Johnson-Laird, 2018). It hardly applies to a deontic modal logic unless you make the utopian assumption that human beings carry out all their obligatory actions. A more plausible assumption is instead that obligatory actions are permissible.

## Modal logics for conditional sentences

Conditional sentences in English, such as:

*If Viv is at the party then Pat is at the party,*

are more complicated than disjunctions, because they have a subordinate clause, their *if*-clause. So, their meanings are harder to pin down (Nickerson, 2015). In the sentential calculus, their logical counterparts are true in any case except the one in which their *if*-clause is true and their *then*-clause is false. This semantics – another instance of a “truth-functional” one – has bizarre consequences, for example, a conditional is true provided that its *if*-clause is false or its *then*-clause is true. And the truth-functional semantics does not apply to counterfactual conditionals such as, granted that Viv is not here:

- *If Viv had been here then Pat would have been here.*

The *if*-clause in this counterfactual interpretation is false, as the preceding set up establishes, and so the standard truth-functional semantics guarantees the truth of the conditional. Yet it could be false, i.e., Viv’s presence might have failed to guarantee Pat’s presence. So the truth-functional semantics is wrong for counterfactuals. The philosopher Grice (1989) made an uncompromising defence of standard logic, and argued that those who reject its semantics overlook the role of the conventions of discourse. But he says nothing about counterfactuals. Two non-standard logics – to which we turn – deal with them.

As a result of Adams’s (1970) argument, the late David Lewis (1973) argued that the standard sentential logic applies to indicative conditionals, whereas a non-standard modal logic with a possible-worlds semantics applies to counterfactuals (see section in the main text on presupposed possibilities and conditionals). Stalnaker (1968) had already proposed such a theory both for factual and counterfactual conditionals. His starting point was Ramsey’s (1990/1929, p. 155) account, not of the meaning of conditionals, which he took to be truth-functional, but of how individuals determine their degree of belief in a conditional. In a famous footnote, he suggested that they add the conditional’s *if*-clause to their knowledge, and then assess their belief in its *then*-clause. In case they believe the *if*-clause, their degree of belief in the conditional depends only on their belief in its *then*-clause. In case they disbelieve the *if-clause*, their degree of belief in the conditional is void. Stalnaker converted this account into a theory of the meaning of conditionals. A conditional is true if and only if its *then*-clause is true in the possible world in which its *if*-clause is true and which otherwise differs minimally from the actual world. When the *if*-clause is false in the actual world, a possible world in which it is true is bound to differ from the actual world When the *if*-clause is true in the actual world, the truth of the conditional depends only on whether its *then*-clause is also true there. And when the *if*-clause is a self-contradiction, the *then*-clause is evaluated in a special “absurd” world in which all contradictions are true. Lewis’s theory (1973) is similar, except that it applies only to counterfactual interpretations of conditionals. It specifies that a counterfactual conditional is true provided that either there is no possible world in which its *if*-clause holds because it is a self-contradiction, or else both its *if-*clause and *then-*clause hold in a possible world that is more similar to the actual world than any possible world in which its *if-*clause holds but its *then*-clause does not hold (for the history of such conditional logics, see Priest, 2008, Ch. 5).

There are many other modal logics, and it is easy to prove that a countable infinity of standard ones exists. One proof (due to Ragni & Johnson-Laird, 2018) rests on the iteration of modal operators, as in this quotation from Grice (1989, p. 65) in which he expresses great tentativeness about a possibility:

*It looks possible (I do not say that it is so, only that it might turn out to be so)...*

So, he took an assertion that “might turn out to be possible” to be weaker than one that “looks possible”. Yet, philosophers can argue that what is possibly possible is no less than possible. Modal inferences in daily life seldom seem to hinge on such matters. Speakers can pile up modal operators solely to be emphatic or to lower the degree of an epistemic possibility, for example, “Perhaps it may be possible that the stock exchange might not collapse”.

## Miniworlds

In its original Leibnizian conception, a possible world determines the truth or falsity of every “atomic” sentence – one without negation, a connective, or a modal expression—in any world to which it is accessible (Kripke, 1963). So each possible world is vast, and so too is the number of them. Each is a *maxiworld* akin to a separate universe, and as the distinguished linguist Partee (1979) wrote, it is too big to fit inside anyone’s head, and calls for a super-competence to assess: “what we would be like if not limited by finite brains and finite experience (e.g., if we were God)”. Indeed, the truth of a claim such as:

*Possibly there are multiple universes*

calls for a possible universe in which is it true. God made us the biggest of all possible worlds to think about.

An alternative logical conception of possibilities restores us to our correct but limited competence. It represents each possibility as a partial description, known as a *model set* (Hintikka, 1967). They are descriptions, not of possible worlds, but of sets of possible worlds, much as mental models represent sets of possibilities. Hintikka’s *model sets* are finite, tractable, and their atomic sentences correspond to recipes for the construction of “pictures” isomorphic to the world. Hintikka relates them to the picture theory of language (Wittgenstein, 1922), and he uses them in his semantics for modal logics. Kripke (1980, p. 15-20) introduced a related idea in his conception of *miniworlds*. He wrote that a possible world is more like a possible state of affairs than a planet, and that possible worlds are akin to textbook descriptions of probability calculations, for example, a throw of two dice yields 6 x 6 = 36 miniworlds. Miniworlds are what model sets describe, but we know of no algorithm that maps modal assertions in English into model sets or miniworlds. Both are comparable to mental models, though they do not distinguish between intuitive and deliberative model sets or miniworlds.

## Appendix 2. The processes that construct mental models and draw conclusions from them

This appendix summarizes the algorithm underlying the theory of mental models as implemented in various computer simulations (Johnson-Laird & Khemlani, 2023): mSentential (at https://www.modeltheory.org/), mReasoner (at https://osf.io/xtrp6/), and mModal (at https://github.com/CognitiveComputationLab/cogmods/tree/master/modal/2019_guerth/models). These programs include a lexicon and a parser that constructs a compositional semantics of sentences, a process that uses a knowledge base to modulate interpretations, and a defeasible component that withdraws a conclusion when a subsequent assertion conflicts with it and uses modulation and its knowledge base to try to resolve the inconsistency. We focus here on modal sentential reasoning based on sentential connectives, the modal operator *possible*, and the use of alethic modals including *necessary* in the evaluation of inferences. We refer readers to Khemlani and Johnson-Laird (2022) for a detailed account of the processes underlying reasoning based on quantified premises. Likewise, we refer them to programs for spatial and temporal reasoning (at https://www.modeltheory.org/) for the processes underlying the construction and manipulation of models of simple sentences without connectives.

The model theory assumes that the mental lexicon contains semantic entries for each connective, such as “and,” “or,” and “if,” that can be used to construct both intuitive and deliberative models. The processes that manipulate the resulting models – to draw conclusions, for example – treat them as representing a conjunction of possibilities that hold in default of knowledge to the contrary, but at least one possibility for a compound must hold for the compound to be true. For example, an exclusive disjunction of the sort:

*Either A or else B, but not both*

where A and B can themselves contain further compounds, has the following two intuitive models:
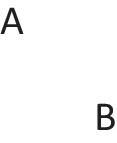


The disjunction has these deliberative models:
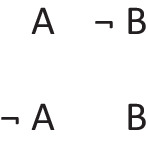


where ‘¬’ symbolizes negation, and has access to its semantics.

In general, negation denies an assertion, and calls for the complement of the set of possibilities to which the assertion refers. For example, the negation of a conjunction, as in:

*Not both A and B*

elicits the complement of the models for *A and B*. This latter affirmative conjunction has only a single model:
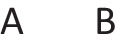


Its complement is therefore:
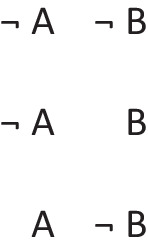


Reasoners have some difficulty in recovering the complete set when they have to enumerate the possibilities to which a negated disjunction refers (Khemlani, Orenes, & Johnson-Laird, 2014).

The models for conditionals reflect the special nature of “if”. As the main text argues, the *if*-clause in *If A then B* presupposes the possibility of *not A*, and so does the negation of the conditional, *If A then not B*. So, a conditional has intuitive models representing its principal possibility and a placeholder for its presupposed possibilities of *not-A*:
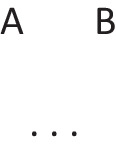


where the ellipsis denotes the placeholder model. It has the corresponding deliberative models in the order of their availability to individuals (see, e.g., Barrouillet & Lecas, 1998):
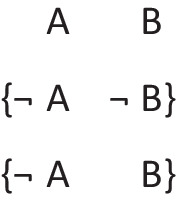


where the brackets demarcate presuppositions, which also hold for the negation of the conditional: *If A then not B* (see Khemlani et al., 2014). It calls for the complement of the preceding models except that presuppositions still hold. So, its intuitive models are:


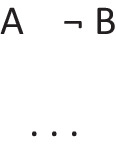


And its deliberative models are:


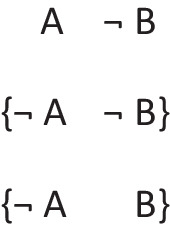


Table 2 summarizes the intuitive and deliberative models for affirmative compounds and their negations based on the meanings of their main connectives.

**Table 2:** The intuitive and deliberative models of affirmative compounds and their negations based on the meanings of their sentential connectives. Each model represents a possibility in default of knowledge to the contrary, and a set of models denotes a conjunction of default possibilities, of which at least one must hold for the truth of the compound

**Name of connective**	**Affirmative assertions**			**Negative assertions**		
	**Compound assertion**	**Intuitive models**	**Deliberative models**	**Negative** **compound**	**Intuitive models**	**Deliberative** **models**
Conjunction	*A and B.*	A B	A B	*Not both A and B.*	¬ A ¬ B . . .	¬ A ¬ B ¬ A B A ¬ B
Exclusive disjunction	*Either A or else B.*	A B	A ¬ B ¬ A B	*Not either A or else B.*	¬ A ¬ B . . .	¬ A ¬ B A B
Inclusive disjunction	*A or B.*	A B A B	A ¬ B ¬ A B A B	*Not(A or B).*	¬ A ¬ B	¬ A ¬ B
Conditional	*If A then B.*	A B . . .	A B {¬ A ¬ B} {¬ A B}	*If A then not B.*	A ¬ B . . .	A ¬ B {¬ A ¬ B} {¬ A B}
Biconditional	*If and only if A then B.*	A B . . .	A B ¬ A ¬ B	*If and only if A then not B.*	A ¬ B . . .	A ¬ B ¬ A B

The symbol ‘¬’ denotes negation, the symbol ‘. . .’ denotes a model with no explicit content standing in for presuppositions, and the symbols ‘{ }’ demarcate the models of presuppositions

Reasoning depends on the ability to form the conjunction of sets of models and to allow the constituents of a compound itself to consist of compounds, and so on recursively. All the required semantic processes can be carried out using only negation and conjunction. Consider the task of conjoining the two sets of deliberative models for this pair of exclusive disjunctions:

- *Either A or else B.*
- *Either B or else C.*

Their two sets of deliberative models presented here side by side are respectively:
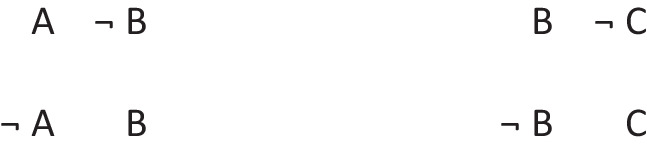


Their pairwise conjunctions call for each consistent pair of models from the two sets to be conjoined into a single model without duplication. The semantics for conjunction (&) carries out the process:


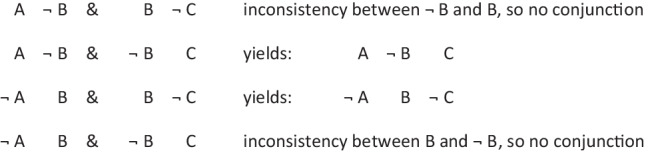


Inconsistency blocks a conjunction, and so the overall result is:
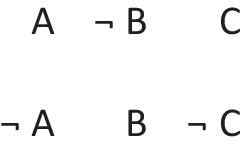


A procedure for drawing conclusions can ignore the “middle” term common to both premises, and conclude, as skilled reasoners tend to do:

*Either A and C or else not A and not C.*

A long-standing algorithm finds the minimal description of a set of models (see, e.g., Johnson-Laird & Byrne, 1991, Ch. 9). It can cope with more complex inferences than naive reasoners can, and it describes the preceding models as: *If and only if A then C*.

The conjunction of two intuitive sets of models is similar except that it has to cope with elements that occur in one model of the pair but not in the other model. We illustrate the process with a compelling fallacy. Individuals tend to judge that the following sorts of pairs of exclusive disjunction are consistent (Khemlani & Johnson-Laird, 2017). But, in fact, they are inconsistent:

*Either A or else B.*

*Either A or else not B.*

Their intuitive models are here side by side:
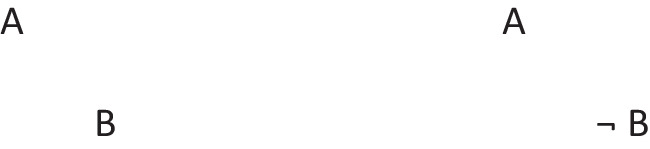


The first step in their conjunction is to combine their respective models on the first lines of each set, with this result, because individuals intuitively conjoin two identical models:
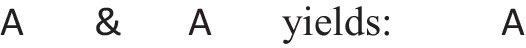


It suffices to judge that the two disjunctions could both be true at the same time. However, the conjunction of each pairing of their deliberative models shows that this judgment is fallacious. The two premises have these respective deliberative models:
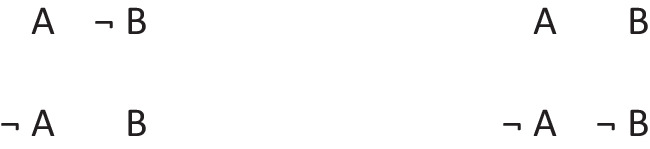


Their conjunction proceeds as follows:
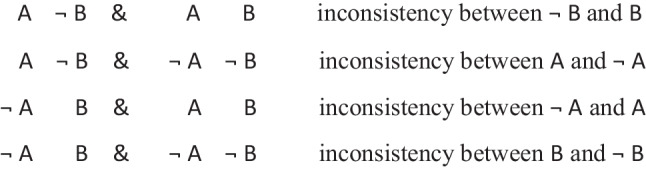


When the conjunction of two sets of models yields an overall inconsistency, it returns the null model, nil, which denotes that there are no possibilities to which the premises refer. The negation of nil yields its complement, T, which denotes a set of models of all possibilities given the premises, and so T is necessarily true. The conjunction of nil with any other model yields nil, and the conjunction of T with any other model yields the other model.

The treatment of a clause that is in turn a compound calls for an appropriate sequence of operations. For example, the following compound:

*Either A and B, or else C*

calls for the models of *A and B* to constitute one clause, *X*, of the exclusive disjunction: *X or else C*, where the value of *X* is the model: A B. The resulting intuitive models are:
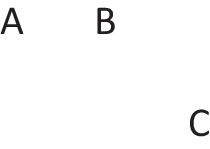


The deliberative models are:
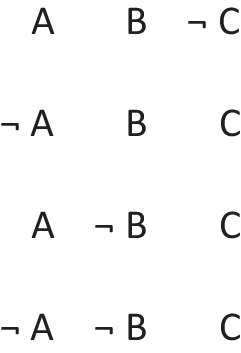


The theory predicts that those reasoners who consider only the intuitive models will infer that the disjunction is inconsistent with the assertion: *A and not-B*. As the deliberative models show, they are wrong. Experimental evidence bears out such illusory fallacies.

The procedure for drawing conclusions from models depends on the alethic relation between the models of the premises and the models of the conclusion. An inference is *necessary* if it preserves, not truth, but possibilities, i.e., its conclusion only refers to at least one possibility or fact to which the premises refer, but reasoners have no need merely to repeat categorical premises as necessary facts (for examples, see the subsection: *correct inferences are alethic necessities*). The computations of the alethic status of an inference calls only for a comparison between the premise models and the conclusion models, because each model refers to what is common to realizations of distinct possibilities.

The construction of models of a premise asserted only to be possible is straightforward. Its intuitive model represent the occurrence of the possibility, and its deliberative model represents the presupposition of its non-occurrence. Consider these premises:

- *Possibly if A then B.*
- *A.*

The intuitive model of the premises represents the occurrence of the conditional and conjoins it with the categorical premise (see Table 1):
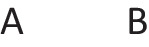


Its deliberative model conjoins the non-occurrence of the conditional with the categorical premise:
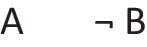


Those individuals who consider only the intuitive model should err and conclude that *B* follows. Only those who consider both models can draw the necessary conclusion:

*Possibly, B.*

Quantified premises of the sort in studies that we report have only one quantifier, and they have a single model representing a set of entities and their properties. The model itself can vary in the number of entities it represents and in its typicality. Consider the following modal premises in which lower case letters denote predicates such as “architects,” “beekeepers,” and “cellists”:

- *Some of the a may be b.*
- *No b is c.*

The single intuitive model represents the possibility to which the first premise refers, and each row represents a different individual, for example:
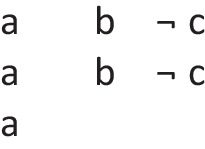


It yields the erroneous categorical conclusion:

$\therefore$ *Some of the a are not c.*

The deliberative model represents that the first premise’s presupposition, i.e., 


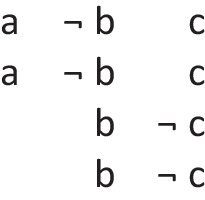


The two models together yield the necessary modal conclusion:
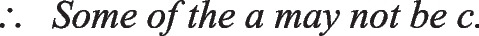


A contrasting pair of premises is:
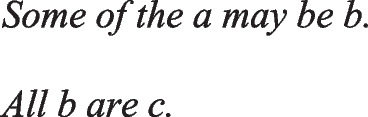


They have an intuitive model in which the possibility occurs, for example:
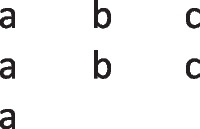


It yields the erroneous categorical conclusion:
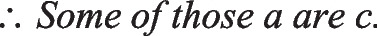


The deliberative model represents the possibility as not occurring:
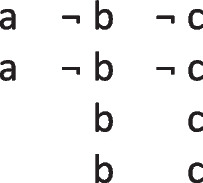


The two models together yield the modal conclusion:
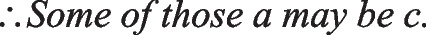


## Supplementary Information

Below is the link to the electronic supplementary material.

Supplementary file1 (DOCX 35 KB)
