# Supplementary material for: Reasoning about possibilities: Modal logics, possible worlds, and mental models
Source: Psychon Bull Rev. 2024 Jul 16;32(1):52–79. doi: 10.3758/s13423-024-02518-z (PMC11836090; doi:10.3758/s13423-024-02518-z)
Supplement: Supplementary file 1 — Supplementary file1 (DOCX 35 KB) [file 13423_2024_2518_MOESM1_ESM.docx]

Cover letter to Editor

Replying to his letter and the reviews	April 4^th^ 2024

To Professor Pennycock, Editor, *Psychonomic Bulletin & Review*

Re ID PBR-TR-24-010: Reasoning about possibilities: modal logics, possible worlds, and mental models

Dear Professor Pennycock,

Thanks again for encouraging us to revise and to resubmit our paper, and for being prepared to make the final decision yourself. We have done our best to meet all the criticisms, and this letter is the detailed one you requested that explains our changes to the paper. Here we reply to in broad terms, but appended are your letter to us and the three reviews with our detailed replies to each point.

We have rewritten the paper with the goal of making it clear—so readers who know nothing about logic or mental models will be able to understand it without too much difficulty. So, every page has been revised, and we have met all substantive points made by Reviewer’s 1 and 2 (see the details below).

Reviewer 3 makes some striking criticisms, and we agree with most of them. The reviewer asks for formally precise definitions. The section of the paper, *Towards a cognitive theory embodying modal logic,* (p. 42), points out the methodological differences between logic and cognitive psychology:

“Logicians call for ‘formally precise’ definitions, and a like-minded reviewer requested a

formal specification of the model theory. Cognitive psychologists do not tend to formulate theories in this way. They aim for a theory that is clear enough both for empirical tests and for computer simulation.”

You will understand that formal precision calls for the use of a symbolic language, and so it is inimical to making the paper easier to understand for those readers with no training in logic.

Also we have presented the definition of ‘validity’ due to a well-known logician just before our definition of ‘necessity’ so that readers can see that both are of a similar degree of informality (p. 18).

Reviewer 3 pointed out that standard modal logics inherit the problems of the sentential calculus. We concur, but they remain problems for modal logics. The reviewer asks what principles leading to difficulty for modal logics concern the model theory’s account of modals, and suspects that there is only one—the condensation of possibilities. In fact, there are four altogether:

1. The aforesaid condensation of possibilities.

2. The model theory postulates that alethic necessity should replace logical validity as the criterion of correct reasoning.

3. The model theory postulates that epistemic possibilities form a scale akin to subjective probability, and that ‘certainty’ is at its top contrary to the treatment in modal logic, which concerns only possibility and necessity, and their respective negations.

4. The model theory postulates that *possibly A* presupposes *possibly* *not-A,* and vice versa. Reviewer 3 states that they can also ‘flip’ in standard modal logics. They can’t. And the revised paper explains why (p. 22):

“Readers might suppose that *possibly A* and *possibly not A* also mutually imply one another in standard modal logics. They do not. The reason is that in all modal logics (except the simplest one, system K), the categorical assertion of *A* implies *possibly A*, and so if the latter implies *possibly not A*, it follows that that *A implies possibly not A*. No modal logic has this implication, because it would lead to absurdities, such as:

*Necessarily it is snowing.*

∴	*Possibly it is snowing.*

∴	*Possibly* *it is not snowing.*

The conclusion contradicts the premise.”

We have edited the paper to deal with reviewer 3’s well-taken criticisms (see the appended details).

We have made many other modifications in the paper to improve its readability, including shifting some arguments from one section of the paper (or the Appendices) to elsewhere in the article. Every page has been edited with the aim of making it easier to understand.

In sum, we are grateful to the reviewers and to you for calling for us to explain the argument of the paper more clearly.

Sincerely,

and on behalf of Phil Johnson-Laird,

Marco Ragni

Here are your letter and the three reviews (in Times New Roman), and our responses to each point in Calibri, and prefaced with: RJL>.

**Editor’s letter**

I have received reviews from three highly qualified reviewers and all suggested revision. Prior to reading the reviews, I also read the manuscript and came to the same conclusion. To summarize, the primary concern is clarity. I’ll admit to also having some difficulty following the manuscript at times, and this is something that I think could hamper the success of the article. Some of that is perhaps inherent to the topic, but more can be done to lower the burden on the reader. The reviewers provide useful feedback in this regard.

RJL> We have tried to be as clear as possible, and followed all the reviewers’ requests for clarity.

In addition to what the reviewers note, I also suggest that the abstract be modified significantly. Right now, it assumes too much background knowledge from the reader. For example, the “two approaches” need to be highlighted more clearly. Also, “modal logic” should be defined in the abstract.

RJL> We have aimed to make the Abstract a stand-alone document requiring no background knowledge, and it now defines ‘modal logic’ and contains everyday examples to illustrate its principal claims.

You’ll note that Reviewer 3 challenges some of the assumptions that are made. Please respond in detail to their comments and add additional argumentation/elaboration to the manuscript where indicated. And, just to flag a particular important request, please make the background knowledge that guides the selection of models more explicit (using the reviewer’s language).

RJL> We have responded to each of Reviewer 3’s challenges, and explained how the theory’s concept of ‘modulation’ accounts for the role of background knowledge in selection of models, using the reviewer’s language where possible (see p. 16).

ACTION: I am rejecting this version of the manuscript, but strongly encouraging a revision if you are able to address the concerns above.

Should you revise the manuscript, I will read the revision to make a decision and only send it out to reviewers if I feel unsure whether these concerns have been adequately addressed. You will receive a reminder 2 weeks before your revision is due.

(Please see instructions below for submitting a revision)

I do not want to have a lengthy process with multiple re-revisions, so I will try to decide on the next version whether the paper should be accepted or not. Thus, please make sure a revision is your best attempt at addressing these issues. If you submit a revision, please include a detailed letter explaining the changes you have made to address the comments here and in the reviews.

RJL> Thanks.

Once again, thank you for submitting your manuscript to Psychonomic Bulletin & Review, and I look forward to receiving your revision.

Sincerely,

Gordon Pennycook

Action Editor, Psychonomic Bulletin & Review

Reviewer 1

The article focuses on how the mental model theory can account for the way people reason

about possibilities, demonstrating that the theory predicts observed phenomena that are at

odds with any standard modal logic. For this purpose, the authors review empirical evidence

for the four main tenets of their theory: correct inferences are necessary, a possibility

presupposes the possibility that it does not occur, this later possibility being represented in a

model when reasoners deliberate, reasoners condense possibilities into one possibility,

inconsistencies have only local effects.

RJL> An accurate summary.

It is a very interesting article that convincingly demonstrates that the mental model theory

accounts far better than alternative explanations for the way people reason about

possibilities. Several examples and supporting experimental findings are provided for each of

the four main tenets listed above. Experimental findings showing that participants endorse

conclusions predicted by the theory, but invalid in standard modal logics, while valid

conclusions in these logics can be rejected when there is no corresponding model, are

especially impressive. The article constitutes an important contribution to human reasoning

that should elicit commentaries and discussions as well as further empirical tests and

theoretical developments. Thus, I think that it represents a perfect match for PBR and

deserves to be published.

RJL> We thank the reviewer for this evaluation.

However, I would like to suggest some minor changes that could make the paper more

impactful and evoke some comments that came to my mind when reading.

- First, the paper is always interesting but particularly dense in some places, especially for

readers, like me, who are not skilled in modal logics. The presentation at the beginning of

the article of the four essentials of standard modal logics is welcome, but it would

sometimes help to rapidly recall in the remaining of the text why some inferences endorsed

by participants are considered as invalid by these logics. For example, on p. 27, the fact that

the paraphrases of indicative and counterfactual conditionals are invalid in standard modal

logics might be not immediate.

RJL> We have reminded readers of the reasons that inferences are invalid throughout the paper, e.g., on pages 8, 16, 31, and 45.

-second, the writing is sometimes so condensed that it became unclear to me what the

authors aim to demonstrate, or what is the conclusions that should be drawn. This for

example the case at the end of the section entitled “Condensation of consistent

possibilities”.

RJL> We have done our best to expand our writing to make it more accessible, including the

section at the end of ‘Condensation of consistent possibilities’.

A more attentive reading could clear up these possible misunderstandings, and it does, but it

would undoubtedly not be useless to help the reader a little. The addition of some

explanations here and there in the text would be welcome. Note that it is additional

evidence for the mental model theory that the conclusions predicted by the theory are far

more immediate and easier to understand by a naive reader than those dictated by the

standard modal logics that are often counterintuitive.

RJL> We take the point that standard modal logics yield conclusions that are counterintuitive, and we have done our best to explain WHY they follow (which bears out the argument of the paper!).

Finally, I would add some comments that do not necessarily call for changes in the paper,

but that the authors could discuss if they find them interesting.

1. On pp. 24-27, the authors refer to studies in which participants produce one or the other

of the two conclusions that the mental model theory predicts. However, I wondered if this

finding is not trivial, because it is difficult to imagine conclusions than those the participants

produced. For example, from “All those architects are cyclist » and « Manuela may be one of

those architects», it is difficult to imagine another conclusion than those favoured by the

participants: “Manuel is a cyclist” or “Manuela may be a cyclist”. The mental model theory

certainly predicts both conclusions depending on the occurrence or not of deliberation, but

is there a theory that would have predicted something else?

RJL>Yes, any theory based on standard modal logic would have predicted something else! At present, we know of no such theory. And unlike the reviewer, we were surprised by how often participants drew categorical conclusions—as the model theory predicts—from premises about possibilities. We cite two studies in which they occur: one carried out in Germany and the other in Portugal.

2. On p. 23, the authors explain that individuals treat the cases in which the antecedent of a

conditional (e.g., if there is a triangle then there is a star) is false (there is no triangle) as not

verifying the conditional because they are also possible for the negation of this same

conditional (if there is a triangle then there is not a star). This is a clever idea, but I doubt

that this is psychologically plausible. For becoming aware that the cases in which there is no

triangle are also possibilities of the negation of the conditional (if there is a triangle then

there is not a star), individual should enter in a complex deliberation, representing what

occurs when the conditional they are evaluating is negated, going beyond the intuitive

model of this conditional (a triangle and no star) to produce deliberative models (no triangle

and no star, no triangle and star), and establishing the correspondence with the deliberative

models of the initial conditional. This constitutes a complex cognitive process, especially

when keeping in mind, as the author note on p. 28 and they are right, that “Mental models

need to be parsimonious given the limited processing capacity of working memory, and so

this principle is a consequence”. Maybe another explanation should be found. This is not

central for the paper, but I have always been concerned with the process of deliberation

(formerly fleshing out), its determinants, constraints, and limits.

RJL> The revised paper describes the reviewer’s misgivings (p. 24):

“A reviewer doubted the psychological plausibility of this account, on the grounds that it

calls for a complex deliberation to grasp the cases hold for both the affirmation and the

denial of a conditional. We agree that such a deliberation would be implausible.”

We go on to explain why it is not necessary for reasoners to make such a deliberation.

3. When discussing Skovgaard-Olsen, Collins, and Klauer (2023), the authors claim, and I

agree, that the model theory does offer an explanation to their results, the split between

“true” and “false” judgments signalling uncertainty within or between participants. It could

be noted that some previous truth-table studies about conditionals reported almost no

“true” or “false” judgments for cases with negated antecedent (see Gauffroy & Barrouillet,

2009, Exp. 1 with NN conditionals in adults). This is probably due to the fact that the option

of response “neither true or false” used by Skovgaard-Olsen et al. was replaced in Gauffroy

and Barrouillet’s study by “one cannot know”. The two options are indeed very different.

The response “neither true or false” refers to a definite third truth value different from

“true” and from “false”, whereas “one cannot know” does not point toward such a third

truth value, but means that cases with a negated antecedent do not convey any information

about the truth value of the conditional. This does not mean that the conditional is “neither

true or false”, something probably difficult to understand by participants; It is certainly true

or flase, but the cases under study do not allow us to know this. Gauffroy and Barrouillet

have demonstrated that these responses (one cannot know) are compatible (and predicted)

by a mental model theory.

RJL> We are grateful to the reviewer for drawing our attention to this result, which we have now incorporated within the paper (see p. 41).

4. It is said in the introduction that the model theory, at least in its beginnings, allowed that

the pragmatic principles of the sort due to Grice were crucial for reasoners’ formulation of

conclusions. I wonder if this is not especially true for modal reasoning. The invalid inference

Possibly B from A or B is so compelling because, pragmatically, there is no sense to utter A or

B when one knows that B is not possible. The mental model theory provides a theoretical

background and the psychological machinery for Grice’s pragmatic principles, but I

wondered if the relationships between the mental model theory and Grice’s intuitions

should not be discussed.

RJL> We cite Grice in several places, but the difficulty in establishing the relation between his account and any psychological theory is that he did not intend his theory to be psychological—he was adamant on this point (in conversation with JL). Moreover, studies of free-choice permission have shown its shortcomings—see p. 33, where we write:

“Gricean theories concern single utterances (Cohen, 1971; see also Bar-Lev & Fox,

2020), and so they cannot explain the following inference, which depends on three assertions:

Your professor told you that you are *permitted* to do only one of the following actions:

*You can do your homework.
You can do the presentation slides.*

∴ *You are permitted to do your homework.*”

Minors.

p. 9 Inconsistency instead of Inconsistent ?

p. 11 “None of the artists”

p. 14 Two occurrences of the word “clauses” in the sentence.

p. 21 They are defaults, i.e., they hold in default

p. 25 ones that participants drew for themselves in a recent study (?)

p. 32: What is the GRE?

p. 38 (E & B) & not (E & B). (?)

condensation of consistent possibilities is invalid. (?)

RJL> Our thanks to the reviewer for spotting these errors. We have corrected all of them.

Reviewer: 2

Comments to the Author

This ms presents a mental model approach to modal reasoning. I do think that this basic approach certainly produces a useful way of understanding how people deal with this kind of reasoning, and I don’t have any specific problems with the ideas presented here. It should be noted that I am not very well versed in the formal kinds of logical models that might be used as a comparison, although I am not very certain that these models are useful when examining how people reason. However, I found this ms to be difficult to follow. The authors often appear to be litigating several debates within the context of their approach and this makes it hard to appreciate the main thread. I would recommend a more straightforward description, which would make it much clearer how this approach can explain actual modal reasoning.

RJL> We have done our best to follow the reviewer’s recommendation to make a straightforward description that focusses on our main point (the inadequacy of modal logics as a guide to human reasoning).

Reviewer: 3

Comments to the Author

This paper compares the mental model theory that elaborates on (cognitively) possible models to draw inferences with (standard) modal logic that is able to model possibility in a rigid logical framework. The authors argue that the four major principles of mental model theory are inconsistent with the four essential principles of modal logic, from which they derive that modal logic is not adequate to provide a proper base for a cognitive theory. They illustrate their claim with many examples.

RJL> We thank the reviewer for an accurate synopsis.

The strength (and also the weakness) of standard (modal) logic is the axiomatic rigidity with which syntax and semantics are linked. In particular, they have a clear semantics that does not, however, generally coincide with the meaning humans associate with an expression. The strength (and also the weakness) of mental model theory is the vagueness with which "models" (not always in the logical sense) are selected and processed to yield inferences. There is no axiomatics, and no clear semantics in mental model theory.

RJL> We accept that our *description* of models may have been vague, but respectfully disagree with the reviewer that the theory itself is vague. It is precise enough to be corroborated in experiments, and implemented in computer programs. See our letter to the editor above.

So, it is quite clear that these two basic approaches are quite diametrically opposed approaches to model reasoning while both have its merits. Since for both modal logics and mental model theory, the "possibility" of a world is a crucial term, comparing them with respect to various (central) aspects is a valuable undertaking and hence, regarding its topic, the paper deserves to be published.

RJL> We are grateful to the reviewer for this recommendation.

However, I found this comparison modal logic vs mental model theory not completely convincing and illuminative. Of course, the paper is a bit biased towards mental model theory because its main evaluation criterion is cognitive adequacy, and it's no secret that standard logics show serious deficiencies here, no doubt. Nevertheless, a  more open and objective comparison would definitely lead to better insights for the main research question of this paper: can a cognitive theory be based on modal logic? (p.4) Or, to be phrased a bit more precisely regarding practical consequences: is there anything that mental model theory can adopt from the modelling of possibility in modal logics, or the other way round?

RJ This point is a good one. We have tried to deal with it in two ways. First, we write in the introduction (p. 3): “Our theory of modal reasoning is based on mental models but it owes much to modal logics, and the present article reciprocates. It presents some ways to modify modal logic so it might serve as a basis for a cognitive theory of reasoning.” Second, these ideas which are designed to help to answer the reviewer’s question: can a cognitive theory be based on modal logic? They are in the penultimate section of the paper titled: ‘Towards a cognitive theory embodying modal logic’ (see p. 42).

First, it should be pointed out clearly that many conflicts that this paper elaborates on are not primarily due to modal logic, but are already present in propositional and first-order logic. Most prominently, the problem with disjunction should be mentioned: From A, infer A or B. This is one of the main point of objection against modal logic, best illustrated by criticizing that from an "exclusive or", an "inclusive or" follows. Of course, that is not intuitive, but modalities have nothing to do with that. This concerns principle 1 (Validity) of modal logic. The same holds for principle 4 (explosive inconsistencies) which is also a problem in propositional and first-order logics. On the positive side of modal logic, principle 1 defines correctness in terms of validity, which is formally precise. What exactly are "correct inferences" in mental model theory? I couldn't find a precise definition here, I think this is implicitly given by necessity, a concept that is quite weak and resembles more a possibility ("one or more of the possibilities or facts to which the premises refer"). As to principles 4 (explosiveness vs. local consequences), here we have the two extremes: in case of an inconsistency, modal logic would infer everything (as all standard logics due, again not due to modalitites) while mental models don't conclude anything, searching for explanations of the conflict instead. Paraconsistent logics might indeed yield better inferences than both modal logics and mental model theory. As to principles 3 (handling of condensation), I couldn't find out what condensation actually is, a precise definition here would be most welcome, and maybe a formal falsification for modal logics, too. Condensation seems to refer to disjunctions, but also examples with conjunctions (p.28) are given.  Regarding principles 2 (possibilities vs impossibilities), I don't see a clash here because, if modal logic implies the possibility of A then it also implies the possibility of not A, and so we can switch between possibility and impossibility. So, nothing to blame on modal logics here. So, in the end, I was not surprized to see that modal logic could not do what mental model theory does (and maybe the other way round?), but regarding my comments to principles 1,2,4, it was not clear to me how this can be justified. Maybe the crucial argument is principle 3 (condensation), but the analysis in the paper is not  clear enough to support any decision on that. A more detailed  and formal elaboration here would be highly appreciated.

RJL> See our letter to the Editor above about the difference between formal logic and cognitive science. Otherwise, the reviewer is correct that modal logics inherit problems of the standard sentential calculus, and we make this point. And in the spirit of the reviewer’s critique we have tried to give clearer definitions and illustrative examples of condensation, and of necessary inferences

The reviewer writes: ‘… if modal logic implies the possibility of A then it also implies the possibility of not A’. No standard modal logic has such an implication: one cannot ‘switch from possibility to impossibility’ in any standard modal logic: see our letter to the editor above for the absurd inferences that follow if one could switch from possibility to impossibility in standard modal logics.

Moreover, a crucial point for relating mental model theory to more standard logics would be to make that background knowledge explicit that guides the selection of models. This is a crucial ingredient to mental model theory, and only if this background knowledge is also represented and used in those logics (as far as possible, e.g., via the reachability relation in modal logics), a fair comparison would be possible. Most interesting would be to find answers to the research question if there is kind of a semantic meta-concept like reachability in modal logic, or similarity between worlds in Lewis' counterfactuals, that guide the selection of models in mental model theory.

RJL> We address the reviewer’s first point on page 16:

“The model theory assumes that knowledge is represented in deliberative models,

but how the process of understanding a premise triggers such models calls for a major research project. In our program simulating modulation, we have used a simplistic process that merely searches for a match between lexical items in a sentence and those in the knowledge base (see mSentential at <https://www.modeltheory.org/models/>). The use of Large Language Models in AI suggests that simple procedures writ large can be surprisingly effective. Once appropriate knowledge is retrieved from long-term memory, a conjunction is made of its set of models with those of the assertion.”

The reviewer’s second point is striking. Possible worlds, as we wrote, are psychologically feasible only if they are the ‘miniworlds’ that Kripke advocated in the second edition or his book on Naming. But, the notion of *similarity* between miniworlds (as used by Stalnaker and Lewis) becomes pretty silly. Miniworlds, Kripke says, are like the 36 possibilities that texts on probability invoke for throws of a pair of dice. Is this miniworld more similar to one for throws of one dice or to one that a person invokes in saying, ‘He may work on one day in the next 36 days’. The best we can say is: it all depends …! But, we agree with the reviewer that an interesting research program would be to examine the psychological analogs of the ‘accessibility’ relations between possible worlds.

Minor and more detailed comments:

p.6, l.3f: Probabilities are not just "possibilities with a numerical measure", their processing follows a clear axiomatic frame whereas the processing of degrees of possibility obeys usually weaker rules; cf. [1].

RJL> We are making a psychological claim, and write:

“The proportions of models supporting a possible conclusion provide a gateway into probabilistic reasoning (Johnson-Laird & Ragni, 2019), which treats subjective probabilities either as non-numerical degrees of intuitive possibility, or, with deliberation, as a numerical scale ranging from, say, 0% to 100% (Khemlani, Lotstein, & Johnson-Laird, 2015).”

.

p.6, l.22: Please explain in more details what "illusionary inferences" are.

RJL> We now write as follows:

“The implementation of the theory in a computer program led to the discovery of compelling and predictable inferences that are fallacious (Johnson-Laird & Savary, 1999)—they seemed at first to reflect a bug in the program, but the bug was in the programmer’s mind. We refer to them as ‘illusory’ inferences, because nearly everyone makes them with great confidence. Yet they are fallacious.” We then give a simple example.

p.14f: Regarding conditionals vs. counterfactuals, please look into the results of empirical evaluations of both with respect to inferences presented in [2], there is a clear difference. However this can be explained, Adams definitely had a point then, so I wouldn't say that his argument is flawed. At least, a more formal elaboration leading in terms of mental models would be necessary here, I couldn't see how this conflict is resolved by mental models.

RJL> We have deleted ‘flawed’ but still point out that Adams’s argument has a hidden confound in it. We deal with the evidence in [2] as follows, which makes a comparison between factual and counterfactual conditionals:

“Byrne and her colleagues have corroborated this account: if an inference depends on the denial of a conditional’s *then*-clause, then individuals are more likely to make it from a counterfactual conditional than from a factual conditional (see, e.g., Byrne, 2017; Byrne & Tasso, 1999; Eichhorn, Kern-Isberner, & Ragni, 2018).”

p.33, l.50: Please explain "GRE".

RJL> We have stated what it stands for: Graduate Record Examination.

p.39, l.17: Shouldn't it be "not (E & B)" instead of "not (E & T)"?

RJL> Yes, we have corrected it.

[1] D. Dubois, J. Lang, and H. Prade. Possibilistic logic. Handbook of Logic in Artificial Intelligence and Logic Programming, D.M. Gabbay, C.H. Hogger, C.H.,  and J.A. Robinson (eds.), Oxford University Press, vol. 3, 1994.

[2] Christian Eichhorn, Gabriele Kern-Isberner, Marco Ragni:

Rational Inference Patterns Based on Conditional Logic. AAAI 2018: 1827-1834
